# Reduction of anion reversal potential subverts the inhibitory control of firing rate in spinal lamina I neurons: towards a biophysical basis for neuropathic pain

**DOI:** 10.1186/1744-8069-2-32

**Published:** 2006-10-13

**Authors:** Steven A Prescott, Terrence J Sejnowski, Yves De Koninck

**Affiliations:** 1 Computational Neurobiology Laboratory, Howard Hughes Medical Institute, Salk Institute for Biological Studies, La Jolla, CA 92037, USA; 2 Division of Biological Sciences, University of California, San Diego, La Jolla, CA 92093, USA; 3 Division de Neurobiologie Cellulaire, Centre de Recherche Université Laval Robert-Giffard, Québec, Québec, Canada G1J 2G3

## Abstract

**Background:**

Reduction of the transmembrane chloride gradient in spinal lamina I neurons contributes to the cellular hyperexcitability producing allodynia and hyperalgesia after peripheral nerve injury. The resultant decrease in anion reversal potential (*i.e*. shift in *E*_anion _to less negative potentials) reduces glycine/GABA_A _receptor-mediated hyperpolarization, but the large increase in membrane conductance caused by inhibitory input can nonetheless shunt concurrent excitatory input. Without knowing the relative contribution of hyperpolarization and shunting to inhibition's modulation of firing rate, it is difficult to predict how much net disinhibition results from reduction of *E*_anion_. We therefore used a biophysically accurate lamina I neuron model to investigate quantitatively how changes in *E*_anion _affect firing rate modulation.

**Results:**

Simulations reveal that even a small reduction of *E*_anion _compromises inhibitory control of firing rate because reduction of *E*_anion _not only decreases glycine/GABA_A _receptor-mediated hyperpolarization, but can also indirectly compromise the capacity of shunting to reduce spiking. The latter effect occurs because shunting-mediated modulation of firing rate depends on a competition between two biophysical phenomena: shunting reduces depolarization, which translates into reduced spiking, but shunting also shortens the membrane time constant, which translates into faster membrane charging and increased spiking; the latter effect predominates when average depolarization is suprathreshold. Disinhibition therefore occurs as both hyperpolarization- and shunting-mediated modulation of firing rate are subverted by reduction of *E*_anion_. Small reductions may be compensated for by increased glycine/GABA_A _receptor-mediated input, but the system decompensates (*i.e*. compensation fails) as reduction of *E*_anion _exceeds a critical value. Hyperexcitability necessarily develops once disinhibition becomes incompensable. Furthermore, compensation by increased glycine/GABA_A _receptor-mediated input introduces instability into the system, rendering it increasingly prone to abrupt decompensation and even paradoxical excitation.

**Conclusion:**

Reduction of *E*_anion _dramatically compromises the inhibitory control of firing rate and, if compensation fails, is likely to contribute to the allodynia and hyperalgesia associated with neuropathic pain. These data help explain the relative intractability of neuropathic pain and illustrate how it is important to choose therapies not only based on disease mechanism, but based on quantitative understanding of that mechanism.

## Background

Neuropathic pain can arise from a multitude of pathophysiologic mechanisms or combinations thereof [1-5]. Reduced inhibition, or disinhibition, of spinal neurons constitutes an important class of these mechanisms [6-8]. Indeed, many features of neuropathic pain syndromes can be reproduced by pharmacologically blocking inhibition in the spinal cord [9-17] or through genetic changes that reduce inhibition [18]. Conversely, increasing inhibition can, in some conditions, reduce neuropathic pain [17,19-22].

Reproducing features of neuropathic pain by reducing inhibition and relieving neuropathic pain by increasing inhibition are important observations but constitute only circumstantial evidence that disinhibition is involved in the pathogenesis of neuropathic pain. More direct evidence comes from studies showing that neuropathy can occlude the effects of pharmacologically reducing inhibition [19] and that inhibition is indeed reduced in animal models of neuropathic pain [23-32]. There is controversy whether reduction of inhibitory transmitters and/or their receptors occurs following neuropathy [33-36], but disinhibition can arise from a broad array of mechanisms.

The recent study by Coull et al. [31] suggests an alternative mechanism to explain disinhibition: reduced expression of the potassium-chloride cotransporter (KCC2) causes reduction of the chloride gradient across the neuronal membrane, which in turn leads to reduction of the anion reversal potential (*i.e. E*_anion _shifts to a less negative membrane potential) [see also [37]]. The change in driving force means that both glycine and GABA_A _receptor-mediated inputs produce less hyperpolarization and could even paradoxically depolarize the neuron. But even if those inputs become depolarizing on their own, their large conductances mean they may still reduce the depolarization caused by concurrent excitatory input [e.g. [38,39]] – a phenomenon known as shunting.

Without knowing the relative contribution of shunting and hyperpolarization to firing rate modulation, it is difficult to predict how reduction of *E*_anion _will impact inhibitory control of firing rate, especially given the nonlinearity inherent in spike generation [40,41]. Recent work has revealed a good correlation between *E*_anion _and pain threshold [42], but it remains unproven whether reductions in *E*_anion _could compromise inhibition sufficiently to produce the cellular hyperexcitability that may in turn cause the perceptual/behavioral features of neuropathic pain including allodynia (perception of pain in response to normally innocuous stimulation) and hyperalgesia (exaggerated pain perception in response to noxious stimulation).

Allodynia and hyperalgesia are typically thought to arise from hyperexcitability at the cellular and network levels [43]. It is implicit in our analysis that responses in lamina I neurons correlate with pain perception, and that hyperexcitability amongst those neurons could therefore give rise to allodynia and hyperalgesia. Although there is no doubt that lamina I neurons convey information to supraspinal targets [44,45], it has been widely assumed that wide dynamic range cells in lamina V are solely capable of encoding stimulus intensity because lamina I cells are predominantly nociceptive specific (which is mistakenly taken to imply that they lack the capacity to modulate their response magnitude depending on stimulus intensity) and that lamina I cells do not receive low threshold input [for review see [46]]. On the contrary, multiple studies have demonstrated the capacity of lamina I projection neurons to encode stimulus intensity [47-51]; moreover, more recent work shows that the A and C fiber nociceptors that innervate lamina I can encode stimulus intensity [52]. It has also been shown that lamina I neurons receive low threshold inputs via polysynaptic pathways, but transmission through those pathways is normally suppressed by inhibition [53]. The cumulated evidence therefore indicates that lamina I projection neurons can encode nociceptive information relevant for pain perception. It logically follows that hyperexcitability of lamina I neurons may contribute to allodynia and hyperalgesia.

This study was undertaken to investigate quantitatively whether reductions in *E*_anion _could compromise inhibition sufficiently to produce cellular hyperexcitability despite compensatory changes that may develop (*e.g*. enhanced GABA_A _components to synaptic events [31]). To this end, we developed a biophysically accurate neuron model for quantitative testing under a variety of conditions. Results demonstrate that even a small reduction of *E*_anion _can cause disinhibition. But whereas compensatory changes may prevent disinhibition resulting from a small reduction of *E*_anion_, reduction of *E*_anion _at magnitudes reported by Coull et al. [31] almost certainly causes incompensable disinhibition. Disinhibition becomes incompensable when compensatory mechanisms fail; the failure of compensation means that the system decompensates and necessarily becomes hyperexcitable. Demonstration that decompensation can occur abruptly, especially in a highly compensated system, may help explain the sometimes unpredictable efficacy of therapeutic interventions and has implications for how to optimize treatment of neuropathic pain.

## Results

### Impact of *E*_anion _on the inhibitory control of firing rate

**Figure 1:**
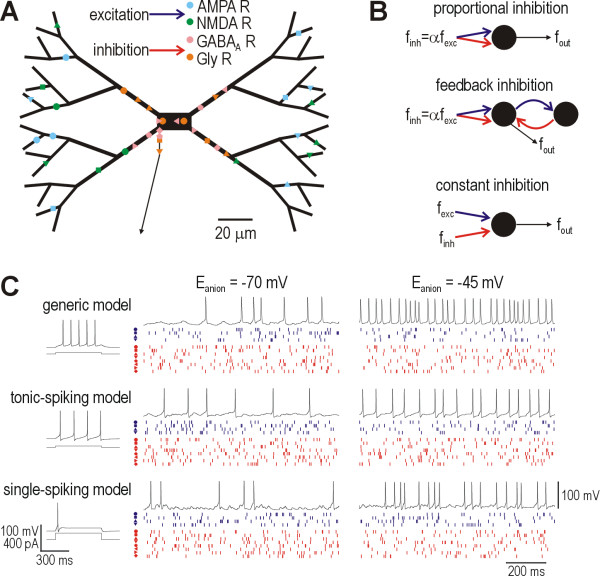
**The model neuron and its synaptic connectivity**. **(A) **The model neuron comprises a soma, 60 dendritic compartments, and an axon; only the most proximal section of the axon is illustrated. Sites of synaptic inputs are shown for conditions corresponding to perisomatic inhibition; another, more uniform distribution of inhibitory synapses was tested (see Methods) but is not illustrated here. Each symbol (circle, square, etc.) denotes membership to a different set of excitatory or inhibitory synapses; synapses in each set receive common input. **(B) **These panels explain the synaptic connectivity responsible for inhibition. Firing rate in the output neuron is denoted *f*_out_. With proportional inhibition, the rate of inhibitory input (*f*_inh_) is proportional to the rate of excitatory input (*f*_exc_) with a constant of proportionality α. With feedback inhibition, the output neuron, which itself receives both excitatory and inhibitory input, excites a feedback neuron that inhibits the output neuron. Since the feedback neuron has the same intrinsic properties as the output neuron, and since a spike in the latter typically elicits a spike in the former, firing rate in the feedback neuron is roughly equal to that in the output neuron. With constant inhibition, *f*_inh _is independent of *f*_exc_. **(C) **Sample responses are shown for each of the three sets of intrinsic membrane properties tested. Panel immediately below each label depicts the response of the model neuron to a 500 ms-long current step injected into the soma. Other panels show responses to random synaptic input (*f*_exc _= *f*_inh _= 80 Hz) for *E*_anion _= -70 mV (left) and -45 mV (right). The voltage response in the model neuron is shown together with the timings of synaptic events in each set of synapses; symbols for each synaptic set correspond to those in part A while color is simply dark blue (excitation) or red (inhibition) because some synaptic sets have more than one type of synapse (*e.g*. AMPA and NMDA).

A multicompartment model based on available data on lamina I neurons was developed as outlined in the Methods (Fig. 1A). The model neuron was bombarded by synaptic input simulated in a biophysically realistic manner: discrete synaptic events generated inward or outward currents by opening channels in the cell membrane (rather than by simply injecting current) so that synaptic inputs influence the total membrane conductance of the postsynaptic neuron. Simulating synaptic input in this way is crucial for uncovering the role of shunting in firing rate modulation. Three varieties of inhibitory input were tested (Fig. 1B): proportional (to excitation), constant, and feedback. We also tested model neurons with different intrinsic properties (Fig. 1C): basic model containing only fast Na^+ ^and delayed rectifier K^+ ^channels (*i.e*. Hodgkin-Huxley or HH channels), tonic-spiking, and single-spiking. Tonic- and single-spiking neurons represent the opposite extremes of physiological cell types in lamina I [54]; their responses to somatic injection of constant current are illustrated in Fig. 1C. Using these models, a systematic investigation of the effects of reducing *E*_anion _on firing rate modulation was carried out.

**Figure 2:**
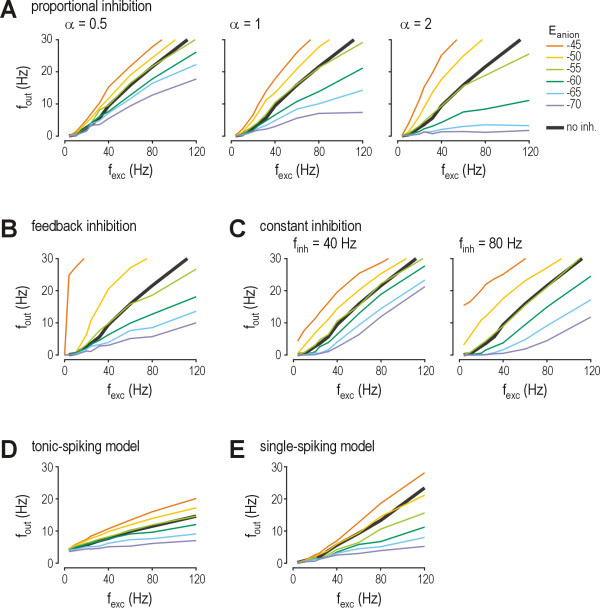
**Reduction of *E*_anion _compromises inhibitory control of firing rate**. Output firing rate (*f*_out_) is plotted against the total rate of EPSPs received from all presynaptic neurons (*f*_exc_) for different values of *E*_anion _tested at 5 mV increments from -70 mV (purple) to -45 mV (orange). The *f*_out_-*f*_exc _curve for no inhibition (α = 0) is shown as a thick black line on each panel. Parts A-C are based on simulations in the basic model. **(A) **With proportional inhibition, *f*_inh _= α *f*_exc_. Each panel shows results for a different value of α. Divergence of the colored curves increases as α increases. **(B) **Feedback inhibition was added to proportional inhibition with α = 0.5. Incorporating feedback inhibition had much the same effect as increasing α under conditions with pure proportional inhibition (see part A) except that, with feedback inhibition, the increased divergence of the colored *f*_out_-*f*_exc _curve was particularly pronounced for *E*_anion _= -50 and -45 mV. This is because those values of *E*_anion _cause paradoxical excitation, meaning feedback inhibition actually becomes feedback excitation (*i.e*. a positive feedback loop), which makes for an extremely hyperexcitable system. **(C) **The final two panels show constant inhibition (*i.e. f*_inh _is independent of *f*_exc_). Under these conditions, the *f*_out_-*f*_exc _curves tend to remain parallel rather than diverge with increasing *f*_exc_, but increasing *f*_inh _nonetheless increases the vertical spacing of those curves. Comparing across parts A-C shows that reduction of *E*_anion _has a similar effect on inhibitory control of firing rate for all three conditions. The *f*_out_-*f*_exc _curves for *E*_anion _= -50 and -45 mV (yellow and orange) exhibit paradoxical excitation since they lie above the *f*_out_-*f*_exc _curve for no inhibition (black). The *f*_out_-*f*_exc _curve for *E*_anion _= -55 mV (light green) exhibits complete disinhibition since it lies very close to the *f*_out_-*f*_exc _curve for no inhibition. The *f*_out_-*f*_exc _curves for *E*_anion _= -60 and -65 mV (dark green and blue) exhibit modest disinhibition since they lie below the *f*_out_-*f*_exc _curve for no inhibition but above the *f*_out_-*f*_exc _curve for *E*_anion _= -70 mV (purple). Based on proportional inhibition with α = 1, simulations were repeated in the tonic-spiking model **(D) **and in the single-spiking model **(E)**. Although the *f*_out_-*f*_exc _curves vary between cell types (compare also with basic model in part A), the more important comparison is between curves for different *E*_anion _within a specific cell type: in the basic and tonic-spiking models, reduction of *E*_anion _to -55 mV causes complete disinhibition, while complete disinhibition in the single-spiking model seems to require a slightly greater reduction, to around -50 mV.

Starting with the basic model neuron, a series of simulations was performed in which the frequency of excitatory synaptic input (*f*_exc_), the frequency of inhibitory synaptic input (*f*_inh_), and *E*_anion _were systematically varied while measuring the frequency of output spiking (*f*_out_). We first posited that both *f*_exc _and *f*_inh _increase with increasingly strong stimulation, and that the increase is proportional though not necessarily equal between excitation and inhibition; the ratio of inhibition to excitation (*f*_inh_/*f*_exc_) is reported as α and was tested at multiple values. Figure 2A shows that reduction of *E*_anion _compromises inhibition's capacity to reduce firing rate. The degree to which firing rate modulation is compromised is most readily understood by comparing the *f*_out_-*f*_exc _curves corresponding to different values of *E*_anion _(colored curves, Fig. 2A) against the *f*_out0_-*f*_exc _curve with no inhibition (*i.e*. α = 0; black curve, Fig. 2A). A reduction of *E*_anion _to -55 mV completely incapacitated inhibition, as evidenced by the corresponding *f*_out_-*f*_exc _curve (light green) lying very close to the no inhibition curve. Reduction of *E*_anion _to -50 or -45 mV caused paradoxical excitation, as evidenced by the corresponding *f*_out_-*f*_exc _curves (yellow and orange) lying above the no inhibition curve. Even modest reduction of *E*_anion _to -65 or -60 mV caused some disinhibition, as evidenced by the corresponding *f*_out_-*f*_exc _curves (blue and dark green) lying above the curve for *E*_anion _= -70 mV (purple) but below the no inhibition curve. The transition from modest disinhibition to complete disinhibition to paradoxical excitation as *E*_anion _was shifted from -70 mV to -45 mV occurred regardless of the ratio of inhibition to excitation (compare panels showing different values of α in Fig. 2A). The divergence the *f*_out_-*f*_exc _curves was, however, exaggerated as α was increased.

Adding feedback inhibition to proportional inhibition with α = 0.5 had an effect (Fig. 2B) similar to increasing the strength of proportional inhibition (*i.e*. increasing α in Fig. 2A). In other words, feedback inhibition exaggerated the effects of reducing *E*_anion_, which was manifested on the graphs as increased divergence of the *f*_out_-*f*_exc _curves. Interestingly, the divergence was more exaggerated for large reductions in *E*_anion _(*i.e*. -45 and -50 mV) compared with small reductions. This is explained by the fact that, when the glycine/GABA_A _receptor-mediated input becomes paradoxically excitatory, a positive feedback loop is born, whereas the feedback loop is negative under normal conditions. Positive feedback (*i.e*. feedback excitation) translates into extreme hyperexcitability.

We then tested the effect of constant inhibition. In contrast to the divergent *f*_out_-*f*_exc _curves seen for proportional inhibition and for feedback inhibition (which is also "proportional" insofar as the feedback neuron's activation is proportional to the output neuron's activity), constant inhibition caused a more parallel shift in the *f*_out_-*f*_exc _curves (Fig. 2C). The vertical separation of those curves was enhanced by increasing *f*_inh_.

Using proportional inhibition, we repeated simulations in a tonic-spiking model neuron (Fig. 2D) and in a single-spiking model neuron (Fig. 2E). While the slopes of the *f*_out_-*f*_exc _curves for these two models were different from those of the basic model (Fig. 2A), the effects of reducing *E*_anion _were very similar: in the tonic-spiking model, reduction of *E*_anion _to -55 mV caused complete disinhibition, the same as in the basic model; the single-spiking model was more resistant, requiring that *E*_anion _be reduced to around -50 mV before disinhibition becomes complete. These data demonstrate that despite the extreme differences in the intrinsic membrane properties of these cell types, firing rate modulation is altered in qualitatively the same way as *E*_anion _becomes reduced.

Data in Figure 2 thus indicate that the degree of reduction in *E*_anion _correlates with the degree to which inhibitory control of spiking is compromised: small reductions (to -65 or -60 mV) cause modest disinhibition, intermediate reductions (to -55 mV) can cause complete disinhibition (*i.e*. equivalent to completely removing inhibition), and large reductions (to -50 or -45 mV) cause paradoxical excitation. This correlation remains quantitatively true regardless of the circuit connectivity (feedback *vs*. proportional inhibition), strength of inhibition (magnitude of α or of *f*_inh_), or intrinsic neuronal properties (tonic- vs. single-spiking). In the more detailed investigation that follows, we focus on proportional inhibition in the basic model but, based on the demonstrations in Figure 2, the results can be reasonably extrapolated to other conditions.

### Relative importance of shunting and hyperpolarization for firing rate modulation

The results above demonstrate that reduction of *E*_anion _significantly compromises inhibitory control of firing rate. But although a reduction in *E*_anion _should logically reduce glycine/GABA_A _receptor-mediated hyperpolarization, a change in *E*_anion _should not reduce glycine/GABA_A _receptor-mediated shunting. The results, therefore, suggest that shunting plays a relatively minor role in the modulation of firing rate, compared with the dominant role of hyperpolarization. This result was unexpected given previous reports on the importance of shunting [e.g. [55,56]] and therefore required further investigation.

**Figure 3:**
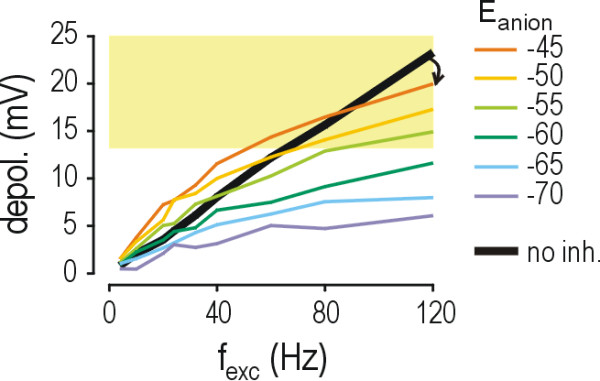
**Shunting has a much greater impact on depolarization than it does on firing rate**. These data are based on simulations in the model neuron with Hodgkin-Huxley (HH) channels removed so as to prevent spiking and thereby allow measurement of the underlying depolarization. Yellow shading shows suprathreshold voltages. α = 1. Unlike the nearly linear *f*_out_-*f*_exc _curves seen in Figure 2, the *depol-f*_exc _curves with inhibition (colored) are clearly sublinear (*i.e*. bend downwards). This sublinearity is not seen in the *depol-f*_exc _curve without inhibition (black). At low *f*_exc_, depolarization is paradoxically enhanced by inhibitory input with *E*_anion _= -50 and -45 mV (*i.e*. the yellow and orange curves lie above the black curve on the left side of the graph) but, because of the sublinearity in those curves, at high *f*_exc_, depolarization is reduced by inhibitory input (*i.e*. the yellow and orange curves lie below the black curve on the right side of the graph; arrow) even if that reduction is less than that for inhibition with *E*_anion _= -70 mV.

The lack of effect of shunting on firing rate contrasts its known effect on depolarization. To investigate in isolation how shunting modulates depolarization, we modified the basic model by removing HH channels, thereby preventing the model neuron from spiking and allowing us to quantify the underlying depolarization. Figure 3 shows that whereas the *depol-f*_exc _curves for *E*_anion _= -45 and -50 mV lay above the curve for no inhibition at low values of *f*_exc_, they bent downwards at higher values of *f*_exc _so that they eventually lay below the curve for no inhibition (arrow in Fig. 3). This sublinearity in the *depol-f*_exc _curves (*i.e*. downward bend with increasing input) is precisely what one would expect from shunting, where increasingly more excitatory input is shunted through open glycine/GABA_A _channels as excitation increases. These results thereby demonstrate that glycine/GABA_A _receptor-mediated shunting remains intact despite reduction of *E*_anion_. As expected, the sublinearity attributable to shunting was absent when there was no inhibition (black *depol-f*_exc _curve in Fig. 3 is linear).

**Figure 4:**
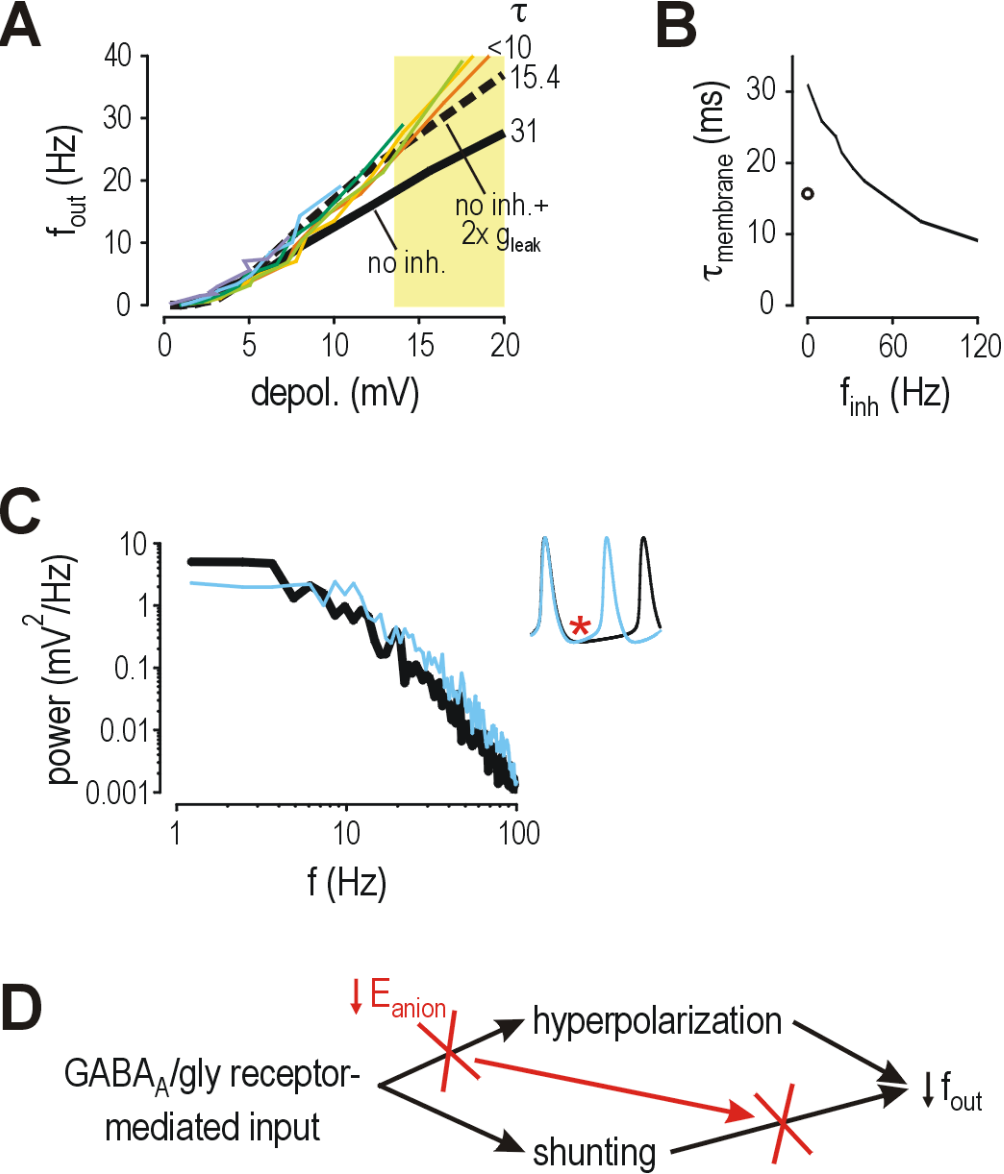
**Reduction of the membrane time constant (τ_membrane_) caused by increased membrane conductance allows for faster spiking**. **(A) **For this graph, *f*_out _produced by a given value of *f*_exc _(based on simulations with HH channels; Fig. 2A, α = 1) was plotted against depolarization produced by the same value of *f*_exc _(based on simulations without HH channels; Fig. 3). The results reveal that, for a given level of depolarization, faster spiking is produced with inhibition than without (compare colored curves with solid black curve). This tendency is unaffected by the value of *E*_anion _and becomes more pronounced with greater depolarization. Yellow shading shows suprathreshold voltages. The increased spiking caused by inhibition is explained by inhibition's reduction of τ_membrane _(see part B); values of τ_membrane _for *depol *≈ 20 mV are shown along right edge of graph. Indeed, if τ_membrane _is reduced to an intermediate value by doubling the passive leak conductance in the model neuron, an intermediate relationship between depolarization and *f*_out _is found (dashed black curve). **(B) **Line shows trend in τ_membrane _as *f*_inh _increases. Dot shows τ_membrane _in model neuron after doubling the passive leak conductance. **(C) **Comparison of power spectra with and without inhibition (blue and black lines, respectively; *f*_inh _= 80 Hz) reveals the reduced low pass filtering that occurs when τ_membrane _is shortened; specifically, frequencies greater than ~7 Hz are associated with higher power when the model neuron is shunted. Inset shows that decreased filtering allows faster membrane recharging between spikes, thereby allowing faster spiking. For this example, stimulus intensity was adjusted to produce equal depolarization (based on simulations without HH channels) with and without inhibition, meaning the difference in interspike interval is attributable solely to a change in τ_membrane_. **(D) **Reduction of *E*_anion _directly compromises glycine/GABA_A _receptor-mediated hyperpolarization. Although shunting itself is unaffected by reduction of *E*_anion_, the ability of shunting to modulate firing rate can be indirectly compromised if, because of reduced glycine/GABA_A _receptor-mediated hyperpolarization, average depolarization remains suprathreshold. In that case, the shunting-induced shortening of τ_membrane _paradoxically yields faster spiking.

But whereas the effects of shunting are clearly evident in the *depol-f*_exc _curves of Figure 3, they are absent from the *f*_out_-*f*_exc _curves of Figure 2 (*i.e*. the *f*_out_-*f*_exc _curves with inhibition are only slightly sublinear). If excitatory input drives depolarization, and depolarization drives spiking, then one should look at the relationship between depolarization and firing rate in order to identify why shunting's capacity to modulate firing rate is lost. To do this, we plotted firing rate (*f*_out_) against depolarization (Fig. 4A) rather than against *f*_exc _(as had been down in Fig. 2). Specifically, we plotted *f*_out _generated by a certain value of *f*_exc _(based on simulations in the model neuron with HH channels) against depolarization generated by the same value of *f*_exc _(based on simulations without HH channels).

### Shunting can paradoxically increase firing rate

Figure 4A reveals a rather counterintuitive observation that, for the same amount of depolarization, significantly faster spiking was generated in the presence of synaptic inhibition than in its absence. This was true regardless of the value of *E*_anion _and became more evident as depolarization increased (see below). The most likely explanation for this is modulation of the membrane time constant (τ_membrane_), since τ_membrane _becomes shorter as membrane conductance increases (Fig. 4B). To test this, we increased the passive leak conductance in the model neuron to reduce τ_membrane _by half (open circle on Fig 4B); like inhibitory synaptic input, increasing the passive leak conductance caused *f*_out _to increase (dashed curve on Fig. 4A) compared with the original model neuron (solid black curve). Why does shortening τ_membrane _lead to faster spiking? When the model neuron was shunted and therefore had a short τ_membrane_, its power spectrum exhibited higher power at all but the lowest frequencies (blue curve in Fig. 4C) compared with the power spectrum for the unshunted neuron with a longer τ_membrane _(black curve). The most important consequence of this reduced filtering of high frequencies is illustrated in the inset of Figure 4C: reduced filtering allows the membrane to recharge more quickly between spikes so that higher firing rates can be achieved when the neuron is shunted than when it is not shunted.

Therefore, shunting can reduce the depolarization caused by excitatory input (Fig. 3), which reduces firing rate (Fig. 4A), but it also shortens τ_membrane _(Fig. 4B), which can increase firing rate (Fig. 4C inset). Both effects occur regardless of the value of *E*_anion_, but their relative importance for firing rate modulation depends on other factors. It is important to recall here that the *depol-f*_exc _curves with inhibition diverge from the *depol-f*_exc _curve without inhibition only when depolarization becomes suprathreshold or, at least, nearly suprathreshold (Fig. 4A). This indicates that that shortening τ_membrane _is only consequential for firing rate modulation when depolarization is suprathreshold, whereas we know from Figure 3 that shunting reduces both sub- and suprathreshold depolarization.

We have previously demonstrated that, when average depolarization is suprathreshold, spikes are generated *deterministically *whereas, when average depolarization is subthreshold, spikes are elicited by noisy, suprathreshold voltage fluctuations and are therefore generated *probabilistic*ally [41]. The rate of probabilistic spiking is reduced by shunting because, by reducing depolarization, shunting increases the difference between average depolarization and voltage threshold, which in turn reduces the likelihood of voltage fluctuations crossing threshold. If shunting can reduce suprathreshold depolarization so that it becomes subthreshold, deterministic spiking will become probabilistic and its rate will be reduced by shunting according to the above mechanism. If, on the other hand, excitation is sufficiently strong to cause net depolarization that remains suprathreshold despite shunting, spiking will be deterministic. Under those conditions, rather than being limited by the probability of threshold crossing, the interspike interval is limited by the rate of interspike membrane charging which, as demonstrated above, is sensitive to both depolarization and τ_membrane_. Shunting reduces depolarization but it also shortens τ_membrane_, where each effect has the opposite impact on the rate of membrane charging. Thus, in regard to firing rate modulation, the dual effects of increased membrane conductance counterbalance each other when spiking is deterministic, whereas the inhibitory effect via reduction of depolarization becomes dominant if spiking is probabilistic. This explanation reconciles the observations in Figures 2 and 3: shunting can reduce depolarization while at the same time having little effect on firing rate.

Since shunting becomes ineffective at modulating firing rate when average depolarization is suprathreshold, one can appreciate that by reducing the hyperpolarization caused by glycine/GABA_A _receptor-mediated input or, worse yet, causing that input to become depolarizing, a reduction of *E*_anion _can indirectly compromise shunting's capacity to reduce firing rate (Fig. 4D). Thus, both mechanisms through which inhibitory input normally modulates firing rate (*i.e*. hyperpolarization and shunting) are both susceptible (directly or indirectly) to changes in *E*_anion_. Furthermore, not only are both inhibitory mechanisms compromised by reduction of *E*_anion_, both mechanisms can contribute to paradoxical excitation if the reduction of *E*_anion _is sufficiently large.

### Effects of constant *vs*. intermittent inhibition

**Figure 5:**
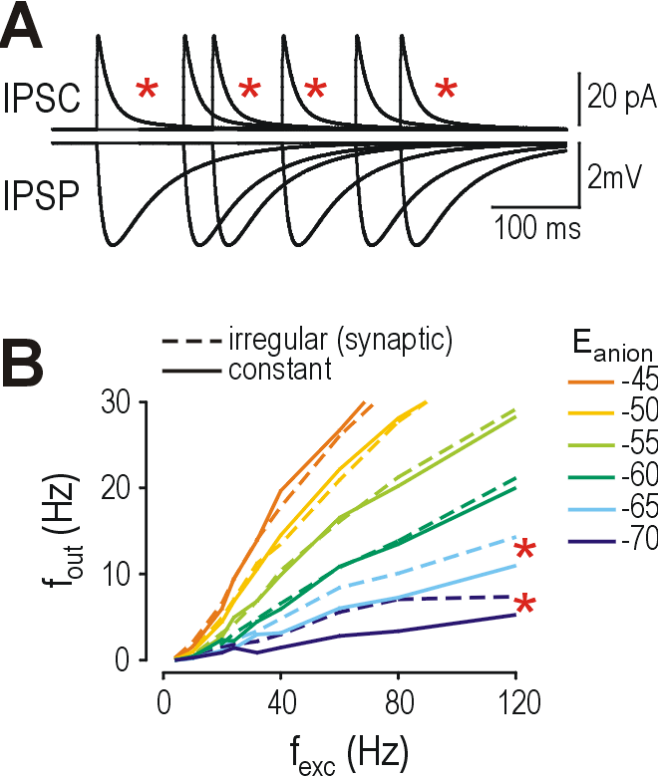
**Gaps in inhibition compromise glycine/GABA_A _receptor-mediated modulation of firing rate only under conditions where shunting can modulate firing rate**. **(A) **Whereas the time course of inhibitory postsynaptic currents (IPSCs) directly parallels the change in membrane conductance, the resultant inhibitory postsynaptic potentials (IPSPs) are much slower because of membrane capacitance. The relative brevity of IPSCs coupled with irregularity in the timing of inputs could allow gaps during which little shunting occurs (red stars). **(B) **Most *f*_out_-*f*_exc _curves were unchanged by switching from intermittent inhibition (dashed lines) to constant inhibition (solid lines); the exceptions were those for *E*_anion _= -65 and -70 mV where constant inhibition caused greater reduction in *f*_out _than intermittent inhibition. This argues in favor of shunting's ability to modulate firing rate only when average depolarization remains subthreshold (see Results). Constant inhibition was applied as a point conductance in the soma equal to the sum of time-averaged conductances from each inhibitory synapse, repeated at each *f*_inh_. α = 1.

We also tested whether the irregularity of inhibitory synaptic input could compromise control of spiking inasmuch as spikes can occur during inhibitory gaps (*i.e*. between inhibitory synaptic events). This irregularity may be expected to compromise modulation of firing rate by shunting more than it compromises modulation by hyperpolarization since inhibitory gaps are larger in the former case. The difference in size of inhibitory gaps is a direct consequence of the differential time course of inhibitory postsynaptic currents (IPSCs) and inhibitory postsynaptic potentials (IPSPs): IPSPs have a slower time course because of the low pass filtering caused by the membrane time constant, so that whereas slow IPSPs tend to overlap and produce sustained potentials, faster IPSCs (which directly parallel the time course of the synaptic conductance) overlap less and, therefore, shunting is likely to be intermittent (Fig. 5A). Quantitative testing confirmed that gaps between IPSCs close more slowly than gaps between IPSPs as *f*_inh _increases (data not shown). To test whether inhibitory gaps are functionally significant for firing rate modulation, we replaced the intermittent inhibition associated with irregular synaptic input with constant inhibition equal to the time-averaged synaptic input.

Figure 5B demonstrates that switching from intermittent to constant inhibition had virtually no effect on *f*_out_-*f*_exc _curves except at the most negative values of *E*_anion_. On initial examination, this suggests that inhibitory gaps are relatively insignificant for firing rate modulation. On closer examination however, the fact that irregular inhibition produces larger gaps in shunting than in hyperpolarization (see above and Fig. 5A) suggests that the irrelevance of switching from intermittent inhibition to constant inhibition may simply reflect the relative impotency of shunting to reduce spiking when spiking is generated in a deterministic manner (see above and Fig. 4). Following from this point, the observation that regularizing inhibition improved the effectiveness of inhibition for *E*_anion _values of -65 and -70 mV (stars in Fig. 5B) is significant, as those *E*_anion _values correspond to the same conditions under which depolarization remained subthreshold at high *f*_exc _(Fig. 3). If average depolarization remains subthreshold, then spiking is generated in a probabilistic manner and shunting should retain it capacity to reduce firing rate (see above). In short, Figure 5B shows that regularizing inhibition enhances shunting's capacity to reduce spiking when spiking is probabilistic, but not when spiking is deterministic, consistent with conclusions drawn from Figure 4 regarding the conditions under which shunting can or can not modulate spiking.

### Disinhibition in the context of compensatory changes and modulation outside lamina I

Reduction of *E*_anion _does not necessarily occur in isolation. The possibility of compensatory changes complicates the conclusion that disinhibition necessarily follows from reduction of *E*_anion_. Two separate studies suggest that GABA_A _transmission may increase following neuropathy [31,57]. We therefore investigated whether disinhibition develops if reduction of *E*_anion _is coupled with compensatory increases in GABA/glycine transmission.

**Figure 6:**
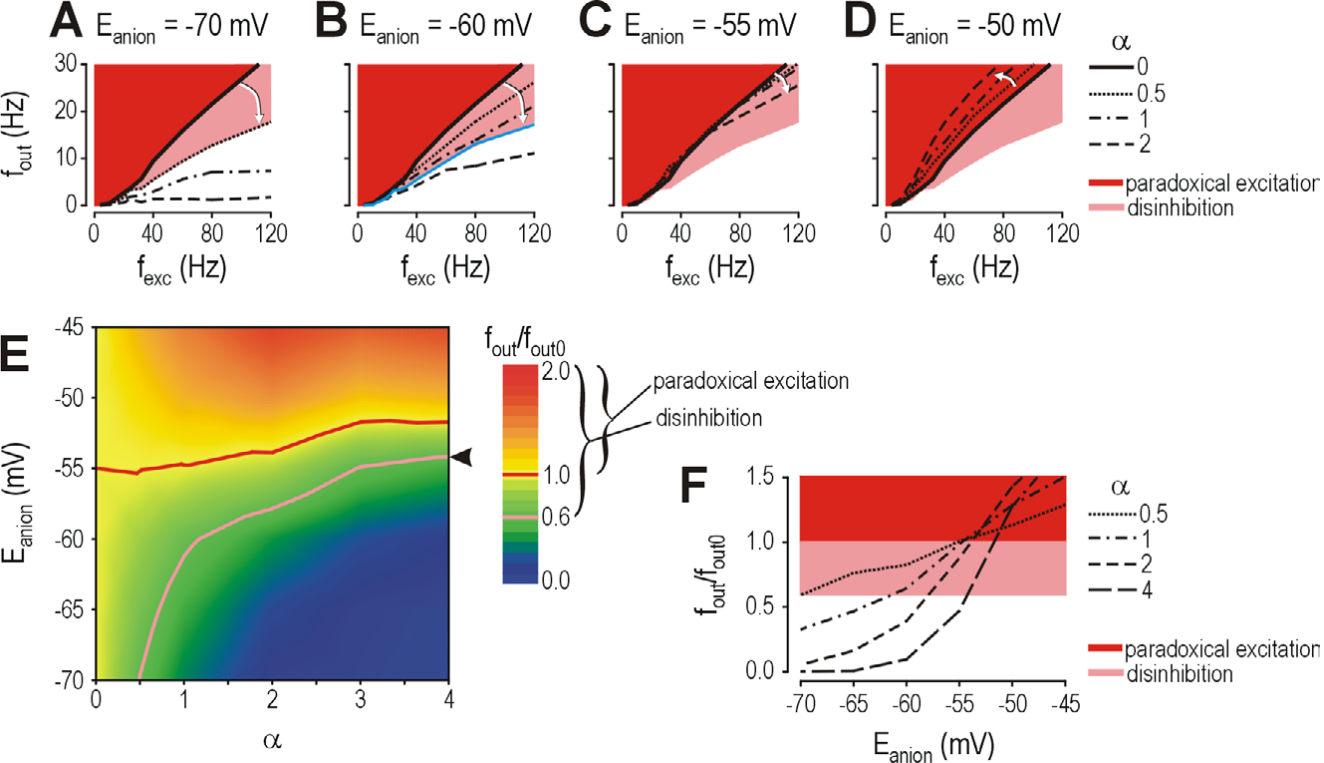
**Compensatory increases in glycine/GABA_A _receptor-mediated input fail to prevent disinhibition if reduction of *E*_anion _exceeds a critical value**. **(A) **Under normal conditions, *E*_anion _= -70 mV. Inhibition with α = 0.5 compresses the *f*_out_-*f*_exc _curve as shown by the white arrow, producing an *f*_out_/*f*_out0 _ratio of approximately 0.6. Reduction of the *f*_out_/*f*_out0 _ratio to 0.6 represents a conservative estimate of inhibition; higher values of α result in greater compression of the *f*_out_-*f*_exc _curve and lower *f*_out_/*f*_out0 _ratios. Using this conservative estimate for illustrative purposes, *f*_out_/*f*_out0 _> 0.6 (pink region) represents disinhibition while *f*_out_/*f*_out0 _> 1 (red region) represents paradoxical excitation. **(B) **If *E*_anion _is reduced to -60 mV, inhibition with α = 0.5 does not reduce *f*_out _as much as it did with *E*_anion _= -70 mV; the resulting *f*_out_-*f*_exc _curve falls inside the pink region, indicative of disinhibition. But if α is increased to 1.2 (blue curve), the *f*_out_-*f*_exc _curve is returned to the white-pink border, meaning *f*_out_/*f*_out0 _≈ 0.6. This demonstrates that disinhibition caused by moderate reduction of *E*_anion _is compensable. **(C) **If *E*_anion _is reduced to -55 mV, increasing α as high as 2 still fails to shift the *f*_out_-*f*_exc _curve outside the pink region, demonstrating that disinhibition caused by larger reduction of *E*_anion _becomes incompensable. **(D) **If *E*_anion _is reduced to -50 mV, increasing α actually shifts the *f*_out_-*f*_exc _curve into the red region, demonstrating that even larger reductions of *E*_anion _can result in paradoxical excitation. **(E) **Contour plot show combinations of *E*_anion _and α that produce disinhibition (*f*_out_/*f*_out0 _> 0.6, demarcated by pink line) and paradoxical excitation (*f*_out_/*f*_out0 _> 1, demarcated by red line) calculated for *f*_exc _= 80 Hz. Arrowheads mark *E*_anion _at which decompensation occurs, assuming α could increase as high as 4. **(F) **These graphs show cross-sections through the contour plots in part E. Increasing α to 4 prevents disinhibition from occurring until *E*_anion _reduces to -54 mV, but steepening of the curve means that decompensation occurs abruptly (*e.g*. reduction of *E*_anion _from -55 to -50 mV causes *f*_out_/*f*_out0 _to nearly triple, increasing from 0.47 to 1.28) whereas disinhibition develops more gradually in the absence of compensation (*e.g*. with α = 0.5, the same change in *E*_anion _causes *f*_out_/*f*_out0 _to change from 1.01 to 1.13).

Under normal conditions with *E*_anion _= -70 mV, even modest amounts of inhibition (*e.g*. α = 0.5) can reduce firing rate (Fig. 6A, dotted curve). The decrease in firing rate can be quantified by taking the ratio of the response with inhibition to the response without inhibition (*f*_out_/*f*_out0_). The *f*_out_/*f*_out0 _ratio is ~0.6 for α = 0.5, but larger values of α result in greater reduction of that ratio (Fig. 6A, dashed and solid curves). For purposes of illustration, we estimate inhibition conservatively (α = 0.5) and, by extension, consider *f*_out_/*f*_out0 _> 0.6 to represent disinhibition. If *E*_anion _were to decrease to -60 mV, inhibition with α = 0.5 would be incapable of reducing *f*_out_/*f*_out0 _to 0.6 (Fig. 6B, dotted curve), meaning disinhibition would occur under those conditions. But if α was increased to 1.2, the *f*_out_/*f*_out0 _ratio would be returned to 0.6 (Fig. 6B, blue curve). Assuming the network has the capacity to at least double α, this demonstrates that moderate reductions in *E*_anion _cause *compensable *disinhibition; in other words, the disinhibition that could potentially result from reduction of *E*_anion _can be compensated for by shifting the balance between excitatory and inhibitory input. However, if *E*_anion _were to decrease to -55 mV, even increasing α to 2 could not reduce *f*_out_/*f*_out0 _to 0.6 (Fig. 6C), demonstrating that disinhibition becomes *incompensable *if reduction of *E*_anion _becomes large. In fact, an increase in α may not only fail to reduce *f*_out_/*f*_out0 _sufficiently (as in Fig. 6C), but may even paradoxically increase *f*_out_/*f*_out0 _as in the case with *E*_anion _= -50 mV (Fig. 6D), resulting in paradoxical excitation. It is interesting to note that paradoxical excitation can occur for values of *E*_anion _below spike threshold, which is approximately -49 mV in the model neuron tested here.

Effects of *E*_anion _on the *f*_out_/*f*_out0 _ratio are summarized in Figure 6E. *f*_out_/*f*_out0 _> 0.6 represents disinhibition based on the conservative estimate of α = 0.5 prior to any compensation, while *f*_out_/*f*_out0 _> 1 represents paradoxical excitation. The relationship between α and the critical value of *E*_anion _beyond which net disinhibition necessarily develops (pink curve on Fig. 6E) shows a ceiling effect wherein increases in α, no matter how large, can not compensate for reductions in *E*_anion _beyond a certain level. For example, assuming α could increase as high as 4, disinhibition would still develop at *E*_anion _≈ -54 mV (arrowhead in Fig. 6E). Stricter limitation on the increase in α would result in disinhibition occurring for even smaller reductions in *E*_anion_.

In a system capable of compensation, reduction of *E*_anion _does not produce disinhibition until the system decompensates; decompensation occurs when reductions in *E*_anion _outstrip compensatory changes. But although compensatory changes may prevent decompensation from occurring until a large reduction of *E*_anion _has developed, Figure 6F shows that in a system relying on strong compensation (*e.g. α *= 4 to maintain an *f*_out_/*f*_out0 _ratio of 0.6), small changes in *E*_anion _may cause large changes in the *f*_out_/*f*_out0 _ratio; in other words, decompensation occurs abruptly, which reflects instability within the system. This is evident from the steepness of the *f*_out_/*f*_out0_-*E*_anion _curve where it passes through *f*_out_/*f*_out0 _= 0.6 (long-dashed curve in Fig. 6F). A system relying on less compensation (*e.g. α *= 1) may decompensate at a lower value of *E*_anion_, but will do so gradually (dot-dashed curve in Fig. 6F), indicating that the system is more stable. Understanding the abruptness of decompensation may help guide therapeutic intervention insofar as it is preferable to reestablish a robust balance between excitation and inhibition rather than an unstable one (see below).

**Figure 7:**
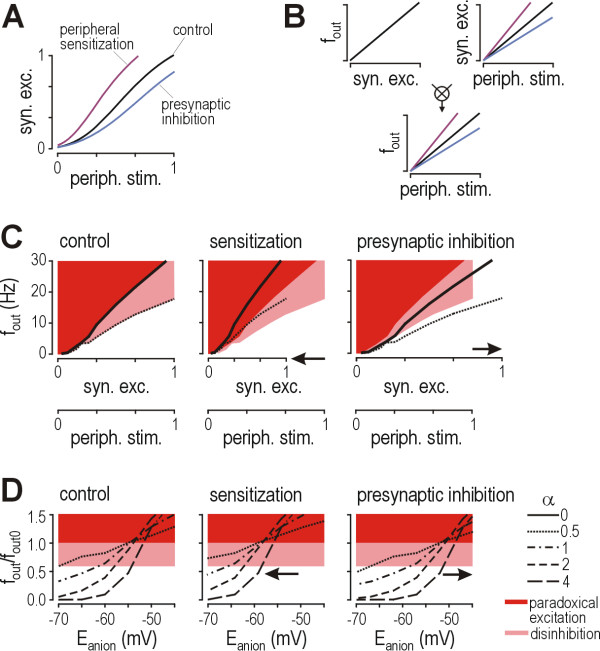
**Peripheral sensitization and presynaptic inhibition alter the amount of postsynaptic inhibition necessary to maintain a normal input-output relationship**. **(A) **Synaptic excitation (*syn. exc*.) of lamina I neurons is assumed to be a sigmoidal function of the strength of peripheral stimulation (*periph. stim*.); both are expressed on an arbitrary scale between 0 and 1. Peripheral sensitization steepens that function whereas presynaptic inhibition flattens it. The distinction between modulation of the frequency or amplitude of synaptic inputs is irrelevant for the analysis here. **(B) **The relationship between *f*_out _and synaptic excitation (which is equivalent to the *f*_out_-*f*_exc _relationship) can be combined with the relationship between synaptic excitation and strength of peripheral stimulation to give the relationship between *f*_out _and strength of peripheral stimulation. **(C) **Peripheral sensitization does not change the relationship between *f*_out _and synaptic excitation, but it does change the relationship between *f*_out _and strength of peripheral stimulation through its effect illustrated in part A. The resulting horizontal compression (left-pointing arrow) forces the *f*_out_-*periph. stim*. curve for α = 0.5 (dotted curve) into the pink region (center panel). This indicates that sensitization has an effect analogous to disinhibition and, by extension, that the neuron must rely on stronger proportional inhibition (*i.e*. larger α) to maintain a normal input-output relationship. Conversely, presynaptic inhibition causes a horizontal expansion (right-pointing arrow) that forces the *f*_out_-*periph. stim*. curve outside the pink region (right panel); under these conditions, the neuron could rely weaker proportional inhibition (i.e. smaller α) to maintain a normal input-output relationship. **(D) **Effects of changing *E*_anion _and α in the context of peripheral sensitization and presynaptic inhibition are illustrated here. The *f*_out_/*f*_out0 _ratio is calculated for *f*_exc _= 80 Hz using *f*_out _for the test condition and *f*_out0 _for the control condition. Thus, *f*_out_/*f*_out0 _> 0.6 represents hyperexcitability comparable to that produced by disinhibition, while *f*_out_/*f*_out0 _> 1 is comparable to hyperexcitability produced by paradoxical excitation. Peripheral sensitization shifts the family of curves leftward (center panel) whereas presynaptic inhibition shifts them rightward (right panel); neither process changes the slopes of those curves, in contrast with the effects of changing α.

Pathophysiologic changes associated with neuropathic pain also occur outside lamina I. How do those changes influence activity in lamina I neurons? We have heretofore assumed a constant relationship between the strength of peripheral stimulation and the strength of synaptic input to lamina I. As a postsynaptic mechanism, disinhibition resulting from reduced *E*_anion _within lamina I neurons does not affect that relationship; on the other hand, presynaptic mechanisms including peripheral sensitization and presynaptic inhibition do (Fig. 7A): peripheral sensitization steepens that relationship by increasing the frequency of excitatory input elicited by a given stimulus, while presynaptic inhibition reduces the amplitude of individual synaptic events. The distinction between modulation of event frequency and event amplitude is irrelevant for this discussion, insofar as cumulative synaptic excitation (in Siemens per second) equals amplitude per input (in Siemens per event) multiplied by event frequency (in events per second); in short, peripheral sensitization increases cumulative synaptic excitation whereas presynaptic inhibition decreases it. Following the simple logic outlined in Figure 7B, the relationship between *f*_out _and synaptic excitation (which for all intents and purposes is equivalent to the *f*_out_-*f*_exc _relationship) can be combined with the relationship between synaptic excitation and strength of peripheral stimulation to determine the relationship between *f*_out _and strength of peripheral stimulation. Figure 7C illustrates how peripheral sensitization and presynaptic inhibition influence the relationship between *f*_out _and peripheral stimulation; note that the relationship between *f*_out _and synaptic excitation remains unchanged. If the *f*_out_/*f*_out0 _ratio is calculated using *f*_out _from the test condition and *f*_out0 _from the control condition, then the effects of peripheral sensitization and presynaptic inhibition can be visualized as leftward or rightward shifts, respectively, in the relationship between *f*_out_/*f*_out0 _and *E*_anion _(Fig. 7D). Thus, whereas peripheral sensitization will exacerbate the effects of postsynaptic disinhibition, presynaptic inhibition will mitigate the effects. Beyond that, reduced pre- or postsynaptic inhibition within polysynaptic pathways may uncover low threshold input to lamina I neurons [53], which would also alter the relationship between the strength of peripheral stimulation and synaptic input.

### Implications for therapeutic interventions

**Figure 8:**
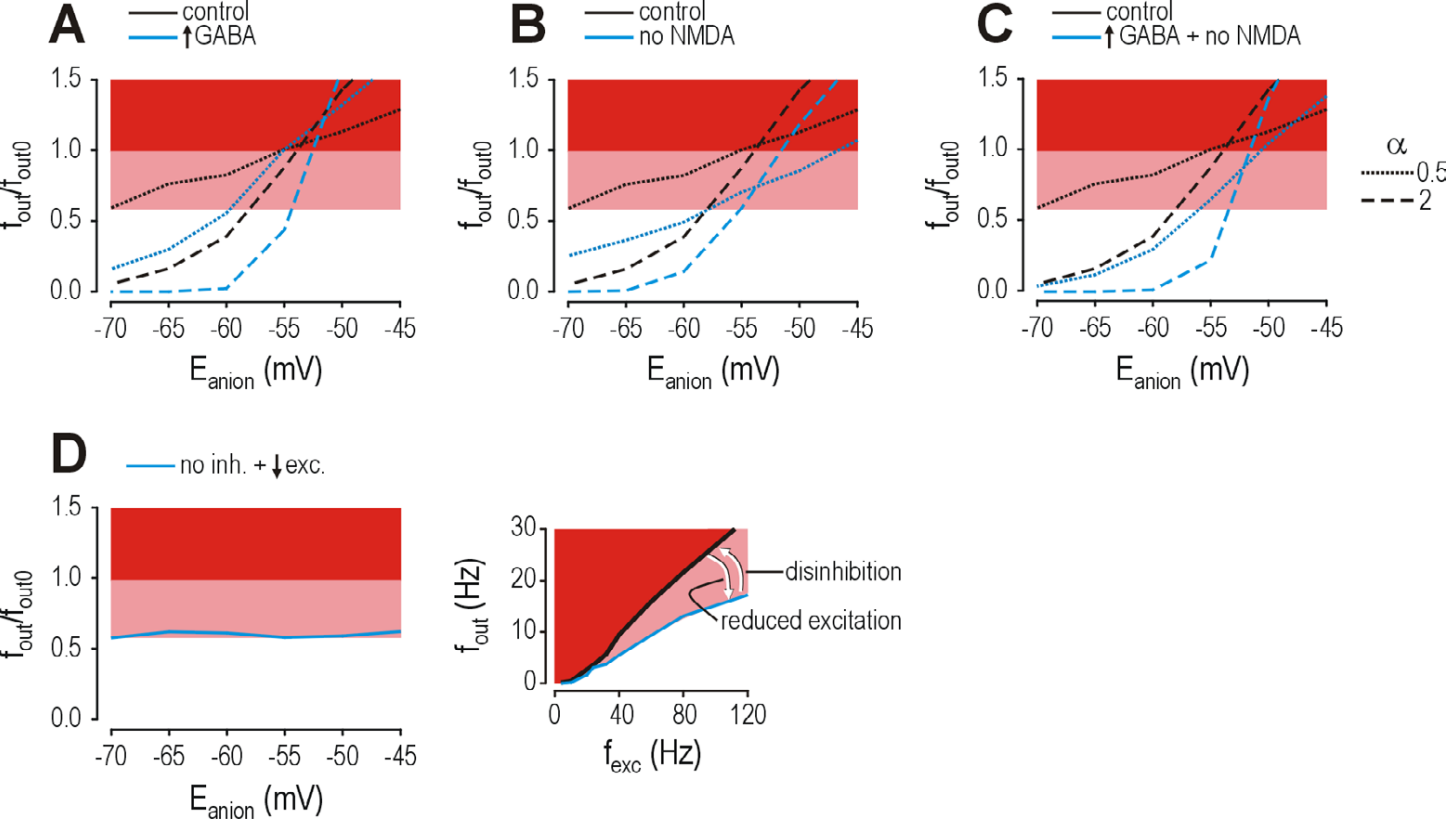
**Therapeutically correcting *E*_anion_-mediated disinhibition by augmenting GABAergic input risks introducing instability into the system, and suggests that other therapeutic interventions may be preferable**. The *f*_out_/*f*_out0 _ratio is calculated for *f*_exc _= 80 Hz. **(A) **Doubling *w *and τ_decay _of GABA_A _receptor-mediated input, as might occur with benzodiazepines, increased the value of *E*_anion _at which decompensation occurred (where curve enters pink region indicating *f*_out_/*f*_out0 _> 0.6), but it risked exacerbating paradoxical excitation if reduction of *E*_anion _was large. Increasing GABAergic transmission had effects comparable to increasing α (compare with Fig. 6F): with α = 0.5 (dotted curve), increasing GABA approximated effects of increasing α to 1.2 (*i.e*. increase of 0.7 or 2.4×) while with α = 2 (dashed curve), increasing GABA approximated effects of increasing α to 4.4 (*i.e*. increase of 2.4 or 2.2×). This demonstrates that strength and frequency of input interact multiplicatively. **(B) **Unlike modulating inhibitory input, blocking NMDA receptor-mediated excitation shifted the curve relating *f*_out_/*f*_out0 _and *f*_exc_. **(C) **Combining NMDA antagonism with increased GABAergic transmission had purely additive effects. Augmenting inhibitory input alone or in combination with reducing excitatory input can prevent decompensation until the reduction in *E*_anion _becomes larger than that necessary to produce decompensation without an increase in inhibition. However, there are several complications: 1) decompensation still occurs for large reduction in *E*_anion_; 2) the balance achieved by increasing inhibition is unstable inasmuch as the curve is steep when passing through *f*_out_/*f*_out0 _= 0.6 meaning small changes in *E*_anion _can cause abrupt decompensation; and 3) for neurons that maintain *E*_anion _= -70 mV, exposure to a benzodiazepine will reduce *f*_out_/*f*_out0 _significantly below 0.6. **(D) **One possible solution to these problems is to deliberately block inhibition (upward arrow in graph on right) and counterbalance the consequent increase *f*_out_/*f*_out0 _by a titrated reduction in excitation (downward arrow). For simulations shown here, GABA, glycine, and NMDA receptor-mediated input were blocked and AMPA receptor-mediated input was decreased until an *f*_out_/*f*_out0 _ratio of ~0.6 was achieved. By removing inhibition, the *f*_out_/*f*_out0 _ratio becomes insensitive to *E*_anion _and α, meaning *f*_out_/*f*_out0 _remains stable despite changes in either variable and, furthermore, variation in *E*_anion _and α between affected and unaffected cells does not influence *f*_out_/*f*_out0_.

It seems logical that if a reduction in *E*_anion _has compromised the strength of inhibition, interventions aimed at reestablishing inhibition's strength would be beneficial for correcting disinhibition. Ideally one would target the primary pathophysiologic cause (*e.g*. KCC2 expression or its direct effects on chloride extrusion from inside the cell, or upstream events including microglial activation and BDNF release [31,42]) but with no clinically available drugs to do this, other targets must be considered. For example, the strength and kinetics of GABA_A _receptor-mediated input can be modulated by benzodiazepines. We therefore investigated the effects of doubling the strength (*w*) and τ_decay _of GABAergic inputs; parameters of glycinergic input were left unchanged. Figure 8A shows that augmenting individual GABAergic inputs mimics the effects of increasing α (compare with Fig. 6F): it delayed decompensation from occurring until higher values of *E*_anion_, but it did so by steepening the curve relating *f*_out_/*f*_out0 _and *E*_anion_. As explained above, this steepening means that although the intervention may rebalance the system at a normal *f*_out_/*f*_out0 _ratio, that balance is liable to be disturbed by even small changes in *E*_anion_; in other words, the system becomes increasingly unstable. Increasing GABAergic transmission also risks exacerbating paradoxical excitation if reduction of *E*_anion _is particularly large. Furthermore, although endogenous compensation may be expected to occur specifically in neurons affected by changes in *E*_anion_, a drug would not show the same specificity, suggesting the *f*_out_/*f*_out0 _ratio would be inadvertently reduced below 0.6 in normal cells (*e.g*. with *E*_anion _= -70 mV) exposed to the drug at the same time that the *f*_out_/*f*_out0 _ratio is potentially normalized in affected cells (*e.g*. with *E*_anion _= -55 mV). Therefore, although a disinhibitory mechanism intuitively suggests that inhibition should be augmented in order to offset the disinhibition, results here suggest that augmenting GABA_A _receptor-mediated input (or, similarly, glycine receptor-mediated input) may be unwise when disinhibition occurs through reduction of *E*_anion_. In contrast, disinhibition occurring through a different mechanism (*e.g*. reduced expression or function of GABA_A _or glycine receptors) could be successfully corrected by augmenting inhibition (see Discussion).

Although increasing inhibition ultimately fails to prevent decompensation if the reduction in *E*_anion _grows too large, and at best reestablishes a normal *f*_out_/*f*_out0 _ratio that is easily disrupted, interventions that do not target inhibition are likely to be more robust. NMDA receptors are a common pharmacological target whose blockade can reduce neuropathic pain [58]. Figure 8B illustrates that blocking NMDA receptors did not alter the slope of the curve (and thus the stability of the system) and instead caused a uniform reduction in the *f*_out_/*f*_out0 _ratio, which is similar to the effect of presynaptic changes described in Figure 7. Furthermore, reducing excitatory input did not risk exacerbating paradoxical excitation. The magnitude of effects of NMDA antagonism depends on the contribution of NMDA receptor-mediated excitation relative to AMPA receptor-mediated excitation, which is relatively small for conditions tested here (see Methods) but may increase under neuropathic conditions [59]. In any case, reducing AMPA receptor-mediated excitation had a similar effect (see below). Reducing sodium channel density, which functionally mimics the postsynaptic effects of local anesthetics and many anti-epileptic medications [60], also resulted in modulation very similar to that described above for NMDA antagonism (data not shown). Insertion of a potassium conductance such as might be activated by opioids [61] also had a similar effect (data not shown). All in all, reducing excitatory input and/or reducing intrinsic neuronal excitability reduces the *f*_out_/*f*_out0 _ratio uniformly across a broad range of *E*_anion_. Combining any of these manipulations with augmentation of inhibition had purely additive effects (Fig. 8C) rather than acting synergistically.

Based on the observation that it may be counterproductive to try to replace inhibition when disinhibition occurs through reduction of *E*_anion_, we explored whether it would be preferable to block inhibitory input altogether and balance the resulting disinhibition with reduction of excitatory input (Fig. 8D). The rationale is that although, on its own, blocking glycine/GABA_A _receptor-mediated input would increase the *f*_out_/*f*_out0 _ratio to 1, the appropriate reduction in excitatory input and/or intrinsic excitability could return the ratio to around 0.6 (Fig. 8D, right panel). The benefit of deliberately switching the disinhibitory mechanism (i.e. from reduction of *E*_anion _to blockade of glycine/GABA_A _receptors) is that blocking glycine/GABA_A _receptor-mediated input would uniformly adjust the *f*_out_/*f*_out0 _ratio to 1, thereby trivializing the variability of *E*_anion _and α between affected and unaffected neurons. Moreover, blocking inhibition would prevent inhibitory input from causing paradoxical excitation in the event that reduction of *E*_anion _was particularly large. One significant requirement is that the distribution and kinetics of the drugs affecting inhibitory and excitatory transmission are similar in order to avoid spatial or temporal mismatches between the two effects. Even if this approach may not be feasible in practice, it illustrates that increasing glycine/GABA_A _receptor-mediated input may be counterproductive in certain neuropathic conditions and that alternative approaches, possibly involving non-intuitive drug combinations, deserve consideration.

## Discussion

This modeling study demonstrates that reduction of *E*_anion _in spinal lamina I neurons can result in disinhibition and hyperexcitability. Compensatory changes may successfully prevent disinhibition and maintain a normal input-output relationship, but they ultimately fail (*i.e*. the system decompensates) if reduction of *E*_anion _exceeds a critical value. Although incompensable disinhibition requires a relatively large reduction in *E*_anion_, the magnitude of that reduction is physiologically plausible and need not be so large as to cause glycine/GABA_A _receptor-mediated inputs to become paradoxically excitatory. Compensatory mechanisms eventually fail because, although glycine/GABA_A _receptor-mediated inputs continue to shunt excitatory inputs despite no longer causing hyperpolarization, the increase in membrane conductance that underlies shunting also shortens the membrane time constant which, under certain circumstances, allows for faster spiking. Deciphering this complex interplay between biophysical mechanisms is, ultimately, important for being able to ascribe perceptual changes (*e.g*. allodynia and hyperalgesia) to pathophysiologic changes at the cellular and molecular level.

### Relative importance of shunting and hyperpolarization for firing rate modulation

Glycine and GABA_A _receptor-mediated inputs are often thought to act via shunting rather than through hyperpolarization given their large conductance and relatively small driving force. Consequently, even depolarizing glycine/GABA_A _receptor-mediated input can have a net inhibitory effect on spike generation by shunting concurrent excitatory input [38,39]. In the current study, we found that shunting was far less significant than hyperpolarization when it comes to modulation of repetitive spiking. This results from increased membrane conductance having opposing effects: it reduces depolarization by shunting excitatory input (thereby decreasing firing rate) but simultaneously shortens the membrane time constant (thereby increasing firing rate). In terms of firing rate modulation, the first effect predominates when average depolarization is subthreshold and spiking is driven by suprathreshold voltage fluctuations (*i.e*. probabilistic spiking) but the two effects offset each other when average depolarization is suprathreshold (*i.e*. deterministic spiking). Shunting therefore becomes ineffective at reducing firing rate when depolarization is suprathreshold, which is especially likely to occur when reduction of *E*_anion _renders glycine/GABA_A _receptor-mediated input less hyperpolarizing or, worse yet, depolarizing. Kuhn et al. [62] have previously described how firing rate modulation is complicated by the dual effects of membrane conductance, noting that average depolarization was reduced by shunting while voltage fluctuations became larger because of the reduced filtering associated with a shortened membrane time constant. Their result applies to probabilistic spiking whereas the effect that we have described applies to deterministic spiking and, in that sense, is distinct.

A recent modeling study that investigated the effects of changes in *E*_anion _on firing rate modulation in a neocortical pyramidal neuron [63] concluded that, because of shunting, GABA remained inhibitory despite changes in *E*_anion_. That would appear to contradict our conclusion that shunting fails in the face of large, but physiologically plausible (as reported in Coull et al. [31]), reductions in *E*_anion_. However, Morita et al. tested only two values of *E*_anion_. According to our evaluation of the phase-planes shown in Figure 4C of their paper, a Hopf bifurcation would not have prevented repetitive spiking if a slightly lower value of *E*_anion _had been tested. There is therefore no discrepancy between our studies but, simply, a difference in the range of *E*_anion _tested.

### Compensable *vs*. incompensable disinhibition

Given this new understanding of the biophysical mechanisms, we can predict whether the disinhibition caused by reduction of *E*_anion _can account for the hyperexcitability that is presumably responsible for the allodynia and hyperalgesia associated with neuropathic pain. We estimate that decompensation would start occurring at *E*_anion _≈ -58 mV (see Fig. 6) assuming that, as compensation, the ratio of inhibitory to excitatory input quadruples relative to the ratio under normal conditions (*i.e*. α increases from 0.5 to 2). Weaker compensation (α increases to only 1) would result in decompensation starting at *E*_anion _≈ -61 mV, whereas stronger compensation (α increases as high as 4) would prevent decompensation until *E*_anion _≈ -54 mV. All of those estimates conservatively assume α = 0.5 under normal conditions (see Results).

Variations in compensation may explain why, with acute manipulations including intrathecal application of BDNF and activated microglia, reduction in *E*_anion _to around -62 mV caused almost an equivalent decrease in withdrawal threshold as that associated with reduction of *E*_anion _to -49 mV following peripheral nerve injury [42]: compensation that could have developed in the latter case, may not have had time to develop in the former case; it is also possible, however, that BDNF has other effects [64] that are unaccounted for in this argument. Additionally, we have not taken into account activity-dependent reduction of the chloride gradient [65-68], meaning a much smaller long-term reduction of *E*_anion _(*i.e*. caused by reduced KCC2 expression) may cause incompensable disinhibition once dynamic, short-term reductions of *E*_anion _(*i.e*. caused by activity-dependent reduction of the chloride gradient) are taken into account. Activity-dependent potassium accumulation is another fast mechanism that may exacerbate reduction of *E*_anion _leading to incompensable disinhibition [69,70]. In any case, given that *E*_anion _reduces on average to -49 mV following peripheral nerve injury [31], the disinhibition that results is most likely incompensable; indeed, the observation that ~20% of lamina I neurons were paradoxically excited by GABA under those conditions [31] suggests that an even larger fraction were incompensably disinhibited (given that disinhibition requires less reduction in *E*_anion _than paradoxical excitation).

Regardless of the precise value of *E*_anion _at which it occurs, incompensable disinhibition approximates the conditions of pharmacologically reduced inhibition, which previous work has shown to be sufficient to produce allodynia and hyperalgesia [9-17]. It logically follows that *incompensable *disinhibition resulting from reduced *E*_anion _is sufficient to explain the exaggerated pain perception associated with neuropathic pain. We could not reach that conclusion if disinhibition were compensable (*i.e*. if compensatory mechanisms could successfully prevent disinhibition) because, in that case, even if the reduction in *E*_anion _could cause allodynia and hyperalgesia, whether or not it did would depend on the success or failure of compensation. Notably, the argument that incompensable disinhibition is sufficient to explain allodynia and hyperalgesia does not exclude other mechanisms from contributing to the aberrant perception; for example, Figure 7 illustrates how peripheral sensitization can exacerbate the hyperexcitability caused by postsynaptic disinhibition. Furthermore, under conditions in which a modest reduction of *E*_anion _occurs, such that some inhibitory capacity remains (e.g. with *E*_anion _<-55 mV), a decrease in GABAergic or glycinergic transmission (either transmitter release or receptor function; see below) would contribute to the disinhibition caused by reduction of *E*_anion_.

### Reduction of E_anion _*vs*. other mechanisms of disinhibition

Although there is general consensus that disinhibition occurs following neuropathy, the mechanism underlying disinhibition has been controversial. Studies have reported that the number of inhibitory neurons in the spinal dorsal horn decreases following peripheral nerve injury [24,26-28], but more recent work has argued against this [34-36]. Other studies have reported a reduction of presynaptic GABA, implicating the GABA transporter GAT1 [32] and the GABA synthesizing enzyme GAD65 [30], but again this has been contradicted by the demonstration of no change in synaptosomal glycine or GABA [33]. In fact, Kontinen et al. [57] reported that GABAergic transmission was increased following neuropathy, presumably as a compensatory change, which would be consistent with the increased GABA contribution to background input to lamina I neurons reported by Coull et al. [31]. Disinhibition through reduction of *E*_anion _does not involve reduced glycinergic or GABAergic transmission but instead works at a downstream locus and controls the potency of inhibitory input. Ironically, although the system has built-in redundancy, inasmuch as it uses both glycine and GABA as fast inhibitory neurotransmitters [71-73], the reduction in *E*_anion _subverts both glycine and GABA_A _receptor-mediated inhibition because of the receptors' common reliance on the transmembrane chloride gradient. This contrasts the mechanism responsible for disinhibition in inflammatory pain, where prostaglandin E2-induced phosphorylation of the glycine receptor decreases glycinergic transmission [74,75] without affecting GABAergic transmission. Under those conditions, increased GABAergic transmission can compensate for decreased glycinergic transmission [76].

In addition to reducing *E*_anion _via decreased KCC2 expression, neuropathy leads to other pathophysiologic changes in lamina I and elsewhere in the pain pathway that undoubtedly contribute to the aberrant perception associated with neuropathic pain [1,3,77-79]. But whereas most other changes perturb a neuron's input-output relationship directly, disinhibition acts indirectly by perturbing a modulatory process. Glycine and GABA_A _receptor-mediated inhibition are crucial mechanisms for the endogenous modulation of pain [80,81], controlling the inflow of Aδ and C fiber-mediated inputs at the segmental level [32,53,82-85] as well as participating in descending modulation originating from the periaqueductal gray and nucleus raphe magnus [86-89]. These control mechanisms are seriously compromised by reduction of *E*_anion_. Disinhibition therefore equates with modulation of a modulatory process, or meta-modulation, and represents a higher order change that not only perturbs the system, but simultaneously compromises the control mechanisms that would otherwise correct that perturbation. This may contribute to the relative intractability of neuropathic pain compared with other types of pain. The ideal therapy would target the primary pathological change and return *E*_anion _to its normal value; this would not only reestablish a normal input-output relationship within the system, but would also restore the system's full capacity to control the input-output relationship. No such therapy currently exists.

### Implications for therapeutics

The mechanism underlying disinhibition does, nonetheless, have significant implications for reestablishing inhibition through therapeutic interventions. Specifically, disinhibition through reduction of *E*_anion _means increasing glycinergic and/or GABAergic transmission may be inconsequential, and potentially even detrimental, depending on the degree of change in *E*_anion_. However, several studies have reported that allodynia was reduced by applying GABA_A _receptor agonists [17,20,22] or by transplantation of GABA-producing cells into the lumbar subarachnoid space [19]. This could be explained by an increase in presynaptic inhibition, since primary afferent terminals do not express KCC2 and are therefore not prone to disinhibition by reduced KCC2 expression [31]. However, the efficacy of increasing GABAergic transmission is controversial: Stubley et al. [21] reported that transplanted-GABA producing cell could prevent allodynia if transplanted early after nerve injury but could not reverse allodynia once it was established, other studies have reported failure of GABA_A _agonists to relieve neuropathic pain after ischemic spinal cord injury [90,91]. These inconsistencies may be attributable to variation in the model of neuropathic pain, but that implicitly assumes that the underlying mechanisms are qualitatively different depending on the model. The present study suggests that variation can also be explained by quantitative differences in the degree of *E*_anion _reduction, *i.e*. whether the system was fully decompensated or whether residual inhibitory capacity remained.

Several authors have advocated the benefits of optimizing the treatment of neuropathic pain by choosing treatments targeted towards mechanisms implicated in the pathogenesis of that pain [92-96]. This study has approached the topic of mechanism-based therapies using quantitative modeling (see Fig. 8). By being quantitative, our results highlight how choosing the optimal therapy might not depend solely on what pathogenic mechanism is involved, but on the degree of change that has occurred, *e.g*. whether the system would benefit from augmenting inhibition depends on how much *E*_anion _is reduced. This may explain the variable efficacy of treatments amongst patients who are suffering from the same neuropathic pain syndrome, where the underlying mechanism is presumably the same but in whom reduction of *E*_anion _may vary.

## Conclusion

Reduction of *E*_anion _can dramatically reduce the inhibitory control of spiking in spinal lamina I neurons, but whether this gives rise to the exaggerated sensitivity characterizing neuropathic pain depends quantitatively on the success or failure of compensatory mechanisms. Understanding the efficacy of therapeutic interventions also depends on this quantitative understanding. These results speak to the importance of using quantitative, biophysically accurate models to bring together the vast panoply of experimental data so that we not only identify mechanisms involved in neuropathic and other types of pain, but so that we understand those mechanisms quantitatively and fully exploit them for clinical benefit.

## Methods

All simulations were performed with NEURON simulation software [97] using a compartmental model of a generic spinal lamina I neuron with resting membrane potential (*V*_rest_) = -63 mV, input resistance (*R*_in_) = 470 MΩ, and membrane time constant (τ_membrane_) = 31 ms, based on average values in Prescott and De Koninck [54] and Coull et al. [31]. Dendrites bifurcated up to fourth order for a total of 60 dendritic compartments (see Fig. 1A). Axial resistivity was 150 Ω·cm. An axon similar to that described by Mainen et al. [98] was attached to the soma. Fast Na^+ ^and delayed rectifier K^+ ^conductances based on Traub and Miles [99] were inserted at 0.1 and 0.01 S/cm^2^, respectively, in the soma, axon initial segment, and axon nodes. Voltage threshold for spiking was approximately -49 mV. A passive leak conductance was distributed evenly throughout the neuron and was adjusted to produce the passive membrane properties described above. Confirmatory testing (see Fig. 2D and 2E) was also performed on two other model neurons with distinct intrinsic properties (Fig. 1C). The tonic-spiking model is the same as model 2 in Figure 9 of our earlier paper [100]. The single-spiking model was derived from the tonic-spiking model by removing *I*_Na, P_, *I*_Ca, P_, *I*_Ca, T_, and *I*_K, S_, and then inserting, at 0.2 mS/cm^2^, a low-threshold K^+ ^current, *I*_K, LT_, modified from the M-type K^+ ^current described in [101]; voltage at half-maximal activation was shifted to -45 mV and kinetics were sped up 100×. For the single-spiking model, all synaptic weights (see below) were tripled in order to overcome the low intrinsic excitability of this cell type.

Synaptic conductances were modeled as a rapid exponential rise in conductance combined with a slower exponential decay in conductance described by τ_rise _and τ_decay_, respectively. Synaptic current is therefore written as *I*_syn_(*t*) = *w *[1-exp(-*t*/τ_rise_)]exp(-*t*/τ_decay_) (*V*_m_-*E*_rev_) where *t *= 0 at the onset of a synaptic event, *w *is synaptic weight, *V*_m _is membrane potential, *E*_rev _is reversal potential, and τ_rise _= 0.5 ms for all synapses. Excitation was mediated by four sets of excitatory synapses distributed throughout the dendrites. Each set consisted of 5 synapses, with 2–3 AMPA synapses and the remainder being NMDA synapses. For AMPA synapses, τ_decay _= 5 ms and *w *was set so that AMPA receptor-mediated events had a peak conductance of 333 pS/synapse [100]. For NMDA synapses, τ_decay _= 25 ms and *w *was left equal to that for AMPA synapses, such that NMDA-receptor mediated events contributed 14% of the total excitatory current in voltage clamp simulations at -60 mV [102]. The voltage-dependent magnesium block of the NMDA current was modeled after Jahr and Stevens [103,104] [see also [105]]. Reversal potential (*E*_rev_) was 0 mV for both AMPA and NMDA receptor-mediated inputs.

Eight sets of 2–3 inhibitory synapses were distributed randomly in the perisomatic region; distributing the inhibitory synapses throughout the dendrites and soma, rather than perisomatically, had no significant effect on the efficacy of inhibition, as confirmed with a separate series of simulations. Half of the inhibitory neuron sets were modeled after glycine synapses with τ_decay _= 12 ms and while the other half were modeled after GABA synapses with τ_decay _= 60 ms [31]. Synaptic weight was adjusted to produce glycine receptor-mediated events with a peak conductance of 450 pS/synapse [72]. Synaptic weight was fivefold less for GABA synapses so that, given the fivefold increase in τ_decay_, GABA receptor-mediated events contributed roughly the same total conductance as glycine receptor-mediated events, as reported by Coull et al. [31]. Mixed glycine/GABA_A _receptor-mediated inhibition simulates the conditions following neuropathy [31], but switching to full glycinergic inhibition did not significantly affect firing rate modulation; results from mixed inhibition are therefore reported throughout. Reversal potential for inhibitory events (which equates with *E*_anion_) was tested at 5 mV increments between -70 mV and -45 mV, which encompasses the range expected for normal and neuropathic conditions [31].

Simulations were intended to mimic the bombardment of a lamina I neuron with input arriving mono- and polysynaptically from multiple primary afferent fibers, where the spike train in each afferent is assumed to be semi-random (*i.e*. the interspike interval distribution has a coefficient of variation (CV) between 0 and 1). The summation of those spike trains gives a cumulative spike train whose distribution has a CV approaching 1 (*i.e*. a Poisson distribution); each set of synapses was therefore driven by an independent Poisson process. For excitatory input, the rate of each Poisson process was multiplied by the number of synaptic sets (*i.e*. 4) so that *f*_exc _specifies the total rate of EPSPs received by the neuron from all presynaptic cells; note that simultaneous activation of multiple excitatory synapses constitutes a single EPSP, which explains why we multiplied by the number of synaptic sets rather than by the total number of synapses in order to calculate the EPSP rate. The EPSP rate reported by Furue et al. [106] using *in vivo *patch clamp falls within the range we tested. Notably, each set of synapses had a slightly different spatial distribution and a different constitution of AMPA and NMDA synapses so that not all primary afferent activity elicited identical EPSPs in the postsynaptic cell. The rate of IPSPs, reported as *f*_inh_, was also calculated by multiplying the Poisson process rate by 4 so that *f*_exc _and *f*_inh _are readily comparable. In keeping with the classical view that excitation is driven mainly by input from small diameter fibers while inhibition is driven mainly by input from large diameter fibers [80], where both types of fibers can be driven by the same peripheral stimulus, we posited that *f*_exc _and *f*_inh _are proportional, although not necessarily equivalent; the constant of proportionality is reported as α, where α = *f*_inh_/*f*_exc_. Since activity in large and small diameter fibers is independent, a temporal relation between EPSPs and IPSPs is not likely to exist, except at the stimulus onset because of the differential conduction velocity in differently sized fibers and the minimum number of intervening synapses (monosynaptic excitation vs. disynaptic inhibition). We have not explicitly modeled the stimulus onset and assume, for proportional inhibition, that excitation and inhibition are temporally independent. The variable delays introduced by signal transmission through polysynaptic pathways encourage this temporal independence, and also support our decision to model inputs as Poisson processes.

In one set of simulations, the time-averaged conductance at each value of *f*_inh _was calculated and subsequently applied as a constant conductance to the soma, thereby replacing the intermittent inhibition produced by irregular synaptic input with constant inhibition. For certain simulations, feedback inhibition was introduced by setting up a simple network (see Fig. 1C) in which the neuron of interest excited another neuron (with the same intrinsic properties as the first) which, in turn, inhibited the neuron of interest. Strength of excitatory synapses was adjusted so that a spike in the presynaptic neuron typically elicited a spike in the postsynaptic neuron, meaning the firing rates of both neurons were roughly equivalent. Based on the delay between spike generation in the output neuron and spike generation in the feedback neuron, and the subsequent synaptic delay, the output neuron experiences an IPSP ~8.5 ms after each spike. Feedback inhibitory synapses were identical to inhibitory synapses described above.

Simulated temperature was 23°C, which is consistent with the kinetics we used here for voltage- and ligand-gated currents. These results can, however, be safely extrapolated to *in vivo *conditions (*i.e*. 37°C). The main concern is that kinetics will be faster at warmer temperatures, which, in the case of inhibitory synaptic input, could result in larger inhibitory gaps. Data in Figure 5 argue that such gaps are relatively unimportant, which is consistent with the absence of any appreciable effect when switching between mixed GABA/glycinergic inhibition and full glycinergic input (see above). All Simulations were 20 s long. With such long simulations, standard deviation for firing rate was only ~0.5 Hz across multiple trials; most conditions were therefore tested with a single trial.

## List of abbreviations

KCC2, potassium-chloride cotransporter 2; *E*_anion_, anion reversal potential; *f*_exc_, rate of excitatory synaptic input; *f*_inh_, rate of inhibitory synaptic input; *f*_out_, rate of output spiking; GABA, γ-aminobutyric acid; HH, Hodgkin-Huxley; IPSC, inhibitory postsynaptic current; IPSP, inhibitory postsynaptic potential; τ_membrane_, membrane time constant.

## Competing interests

The author(s) declare that they have no competing interests.

## Authors' contributions

SAP designed the study and performed all simulations. YDK helped conceive the study. All authors contributed to preparation of the final manuscript.

## References

[B1] Woolf CJ, Salter MW (2000). Neuronal plasticity: increasing the gain in pain. Science.

[B2] Woolf CJ, Mannion RJ (1999). Neuropathic pain: aetiology, symptoms, mechanisms, and management. Lancet.

[B3] Woolf CJ (2004). Dissecting out mechanisms responsible for peripheral neuropathic pain: implications for diagnosis and therapy. Life Sci.

[B4] Koltzenburg M, Scadding J (2001). Neuropathic pain. Curr Opin Neurol.

[B5] Besson JM (1999). The neurobiology of pain. Lancet.

[B6] Dickenson AH (1996). Balances between excitatory and inhibitory events in the spinal cord and chronic pain. Prog Brain Res.

[B7] Wiesenfeld-Hallin Z, Aldskogius H, Grant G, Hao JX, Hokfelt T, Xu XJ (1997). Central inhibitory dysfunctions: mechanisms and clinical implications. Behav Brain Sci.

[B8] Sandkühler J (1996). Neurobiology of spinal nociception: new concepts. Prog Brain Res.

[B9] Yaksh TL (1989). Behavioral and autonomic correlates of the tactile evoked allodynia produced by spinal glycine inhibition: effects of modulatory receptor systems and excitatory amino acid antagonists. Pain.

[B10] Sherman SE, Loomis CW (1994). Morphine insensitive allodynia is produced by intrathecal strychnine in the lightly anesthetized rat. Pain.

[B11] Sivilotti L, Woolf CJ (1994). The contribution of GABA(A) and glycine receptors to central sensitization: disinhibition and touch-evoked allodynia in the spinal cord. J Neurophysiol.

[B12] Sherman SE, Loomis CW (1995). Strychnine-dependent allodynia in the urethane-anesthetized rat is segmentally distributed and prevented by intrathecal glycine and betaine. Can J Physiol Pharmacol.

[B13] Sherman SE, Loomis CW (1996). Strychnine-sensitive modulation is selective for non-noxious somatosensory input in the spinal cord of the rat. Pain.

[B14] Sorkin LS, Puig S (1996). Neuronal model of tactile allodynia produced by spinal strychnine: effects of excitatory amino acid receptor antagonists and a mu-opiate receptor agonist. Pain.

[B15] Sorkin LS, Puig S, Jones DL (1998). Spinal bicuculline produces hypersensitivity of dorsal horn neurons: effects of excitatory amino acid antagonists. Pain.

[B16] Loomis CW, Khandwala H, Osmond G, Hefferan MP (2001). Coadministration of intrathecal strychnine and bicuculline effects synergistic allodynia in the rat: an isobolographic analysis. J Pharmacol Exp Ther.

[B17] Malan TP, Mata HP, Porreca F (2002). Spinal GABA(A) and GABA(B) receptor pharmacology in a rat model of neuropathic pain. Anesthesiology.

[B18] Ugarte SD, Homanics GE, Firestone LL, Hammond DL (2000). Sensory thresholds and the antinociceptive effects of GABA receptor agonists in mice lacking the beta3 subunit of the GABA(A) receptor. Neuroscience.

[B19] Hwang JH, Yaksh TL (1997). The effect of spinal GABA receptor agonists on tactile allodynia in a surgically-induced neuropathic pain model in the rat. Pain.

[B20] Eaton MJ, Plunkett JA, Martinez MA, Lopez T, Karmally S, Cejas P, Whittemore SR (1999). Transplants of neuronal cells bioengineered to synthesize GABA alleviate chronic neuropathic pain. Cell Transplant.

[B21] Stubley LA, Martinez MA, Karmally S, Lopez T, Cejas P, Eaton MJ (2001). Only early intervention with gamma-aminobutyric acid cell therapy is able to reverse neuropathic pain after partial nerve injury. J Neurotrauma.

[B22] Rode F, Jensen DG, Blackburn-Munro G, Bjerrum OJ (2005). Centrally-mediated antinociceptive actions of GABA(A) receptor agonists in the rat spared nerve injury model of neuropathic pain. Eur J Pharmacol.

[B23] Woolf CJ, Wall PD (1982). Chronic peripheral nerve section diminishes the primary afferent A-fibre mediated inhibition of rat dorsal horn neurones. Brain Res.

[B24] Castro-Lopes JM, Tavares I, Coimbra A (1993). GABA decreases in the spinal cord dorsal horn after peripheral neurectomy. Brain Res.

[B25] Stiller CO, Cui JG, O'Connor WT, Brodin E, Meyerson BA, Linderoth B (1996). Release of gamma-aminobutyric acid in the dorsal horn and suppression of tactile allodynia by spinal cord stimulation in mononeuropathic rats. Neurosurgery.

[B26] Ibuki T, Hama AT, Wang XT, Pappas GD, Sagen J (1997). Loss of GABA-immunoreactivity in the spinal dorsal horn of rats with peripheral nerve injury and promotion of recovery by adrenal medullary grafts. Neuroscience.

[B27] Ralston DD, Behbehani M, Sehlhorst SC, Meng XW, Ralston HJ, Jensen TS, Turner JA and Wiesenfeld-Hallin Z (1997). Decreased GABA immunoreactivity in rat dorsal horn is correlated with pain behavior: a light and electron microscope study.

[B28] Eaton MJ, Plunkett JA, Karmally S, Martinez MA, Montanez K (1998). Changes in GAD- and GABA- immunoreactivity in the spinal dorsal horn after peripheral nerve injury and promotion of recovery by lumbar transplant of immortalized serotonergic precursors. J Chem Neuroanat.

[B29] Fukuoka T, Tokunaga A, Kondo E, Miki K, Tachibana T, Noguchi K (1998). Change in mRNAs for neuropeptides and the GABA(A) receptor in dorsal root ganglion neurons in a rat experimental neuropathic pain model. Pain.

[B30] Moore KA, Kohno T, Karchewski LA, Scholz J, Baba H, Woolf CJ (2002). Partial peripheral nerve injury promotes a selective loss of GABAergic inhibition in the superficial dorsal horn of the spinal cord. J Neurosci.

[B31] Coull JA, Boudreau D, Bachand K, Prescott SA, Nault F, Sik A, De Koninck P, De Koninck Y (2003). Trans-synaptic shift in anion gradient in spinal lamina I neurons as a mechanism of neuropathic pain. Nature.

[B32] Miletic G, Draganic P, Pankratz MT, Miletic V (2003). Muscimol prevents long-lasting potentiation of dorsal horn field potentials in rats with chronic constriction injury exhibiting decreased levels of the GABA transporter GAT-1. Pain.

[B33] Somers DL, Clemente FR (2002). Dorsal horn synaptosomal content of aspartate, glutamate, glycine and GABA are differentially altered following chronic constriction injury to the rat sciatic nerve. Neurosci Lett.

[B34] Polgar E, Hughes DI, Riddell JS, Maxwell DJ, Puskar Z, Todd AJ (2003). Selective loss of spinal GABAergic or glycinergic neurons is not necessary for development of thermal hyperalgesia in the chronic constriction injury model of neuropathic pain. Pain.

[B35] Polgar E, Gray S, Riddell JS, Todd AJ (2004). Lack of evidence for significant neuronal loss in laminae I-III of the spinal dorsal horn of the rat in the chronic constriction injury model. Pain.

[B36] Polgar E, Hughes DI, Arham AZ, Todd AJ (2005). Loss of neurons from laminas I-III of the spinal dorsal horn is not required for development of tactile allodynia in the spared nerve injury model of neuropathic pain. J Neurosci.

[B37] Price TJ, Cervero F, De Koninck Y (2005). Role of cation-chloride-cotransporters (CCC) in pain and hyperalgesia. Curr Top Med Chem.

[B38] Staley KJ, Mody I (1992). Shunting of excitatory input to dentate gyrus granule cells by a depolarizing GABA(A) receptor-mediated postsynaptic conductance. J Neurophysiol.

[B39] Gulledge AT, Stuart GJ (2003). Excitatory actions of GABA in the cortex. Neuron.

[B40] Chance FS, Abbott LF, Reyes AD (2002). Gain modulation from background synaptic input. Neuron.

[B41] Prescott SA, De Koninck Y (2003). Gain control of firing rate by shunting inhibition: roles of synaptic noise and dendritic saturation. Proc Natl Acad Sci USA.

[B42] Coull JAM, Beggs S, Boudreau D, Boivin D, Tsuda M, Inoue K, Gravel C, Salter MW, De Koninck Y (2005). BDNF from microglia causes the shift in neuronal anion gradient underlying neuropathic pain. Nature.

[B43] Jensen TS, Baron R (2003). Translation of symptoms and signs into mechanisms in neuropathic pain. Pain.

[B44] Trevino DL, Zotterman Y (1976). The origin and projections of a spinal nociceptive and thermoreceptive pathway. Sensory Functions of the Skin.

[B45] Perl ER, Darian-Smith I (1984). Pain and nociception. Sensory processes.

[B46] Craig AD (2003). Pain mechanisms: labeled lines versus convergence in central processing. Annu Rev Neurosci.

[B47] Craig AD, Kniffki KD (1985). Spinothalamic lumbosacral lamina I cells responsive to skin and muscle stimulation in the cat. J Physiol.

[B48] Bester H, Chapman V, Besson JM, Bernard JF (2000). Physiological properties of the lamina I spinoparabrachial neurons in the rat. J Neurophysiol.

[B49] Craig AD, Krout K, Andrew D (2001). Quantitative response characteristics of thermoreceptive and nociceptive lamina I spinothalamic neurons in the cat. J Neurophysiol.

[B50] Andrew D, Craig AD (2002). Responses of spinothalamic lamina I neurons to maintained noxious mechanical stimulation in the cat. J Neurophysiol.

[B51] Craig AD, Andrew D (2002). Responses of spinothalamic lamina I neurons to repeated brief contact heat stimulation in the cat. J Neurophysiol.

[B52] Slugg RM, Campbell JN, Meyer RA (2004). The population response of A- and C-fiber nociceptors in monkey encodes high-intensity mechanical stimuli. J Neurosci.

[B53] Torsney C, MacDermott AB (2006). Disinhibition opens the gate to pathological pain signaling in superficial neurokinin 1 receptor-expressing neurons in rat spinal cord. J Neurosci.

[B54] Prescott SA, De Koninck Y (2002). Four cell types with distinctive membrane properties and morphologies in lamina I of the spinal dorsal horn of the adult rat. J Physiol.

[B55] Eccles JC (1964). The Physiology of Synapses.

[B56] Borg-Graham LJ, Monier C, Fregnac Y (1998). Visual input evokes transient and strong shunting inhibition in visual cortical neurons. Nature.

[B57] Kontinen VK, Stanfa LC, Basu A, Dickenson AH (2001). Electrophysiologic evidence for increased endogenous GABAergic but not glycinergic inhibitory tone in the rat spinal nerve ligation model of neuropathy. Anesthesiology.

[B58] Chizh BA, Headley PM (2005). NMDA antagonists and neuropathic pain - multiple drug targets and multiple uses. Curr Pharm Des.

[B59] Isaev D, Gerber G, Park SK, Chung JM, Randik M (2000). Facilitation of NMDA-induced currents and Ca2+ transients in the rat substantia gelatinosa neurons after ligation of L5-L6 spinal nerves. Neuroreport.

[B60] Kalso E (2005). Sodium channel blockers in neuropathic pain. Curr Pharm Des.

[B61] Yoshimura M, North RA (1983). Substantia gelatinosa neurones hyperpolarized in vitro by enkephalin. Nature.

[B62] Kuhn A, Aertsen A, Rotter S (2004). Neuronal integration of synaptic input in the fluctuation-driven regime. J Neurosci.

[B63] Morita K, Tsumoto K, Aihara K (2005). Possible effects of depolarizing GABA(A) conductance on the neuronal input-output relationship: a modeling study. J Neurophysiol.

[B64] Pezet S, McMahon SB (2006). Neurotrophins: mediators and modulators of pain. Annu Rev Neurosci.

[B65] Qian N, Sejnowski TJ (1990). When is an inhibitory synapse effective?. Proc Natl Acad Sci USA.

[B66] Staley KJ, Soldo BL, Proctor WR (1995). Ionic mechanisms of neuronal excitation by inhibitory GABA(A) receptors. Science.

[B67] Staley KJ, Proctor WR (1999). Modulation of mammalian dendritic GABA(A) receptor function by the kinetics of Cl- and HCO3- transport. J Physiol.

[B68] Cordero-Erausquin M, Coull JA, Boudreau D, Rolland M, De Koninck Y (2005). Differential maturation of GABA action and anion reversal potential in spinal lamina I neurons: impact of chloride extrusion capacity. J Neurosci.

[B69] Kaila K, Lamsa K, Smirnov S, Taira T, Voipio J (1997). Long-lasting GABA-mediated depolarization evoked by high-frequency stimulation in pyramidal neurons of rat hippocampal slice is attributable to a network-driven, bicarbonate-dependent K+ transient. J Neurosci.

[B70] Jarolimek W, Lewen A, Misgeld U (1999). A furosemide-sensitive K+-Cl- cotransporter counteracts intracellular Cl- accumulation and depletion in cultured rat midbrain neurons. J Neurosci.

[B71] Todd AJ (1996). GABA and glycine in synaptic glomeruli of the rat spinal dorsal horn. Eur J Neurosci.

[B72] Chéry N, De Koninck Y (1999). Junctional versus extrajunctional glycine and GABA(A) receptor-mediated IPSCs in identified lamina I neurons of the adult rat spinal cord. J Neurosci.

[B73] Keller AF, Coull JA, Chery N, Poisbeau P, De Koninck Y (2001). Region-specific developmental specialization of GABA-glycine cosynapses in laminas I-II of the rat spinal dorsal horn. J Neurosci.

[B74] Harvey RJ, Depner UB, Wassle H, Ahmadi S, Heindl C, Reinold H, Smart TG, Harvey K, Schutz B, Abo-Salem OM, Zimmer A, Poisbeau P, Welzl H, Wolfer DP, Betz H, Zeilhofer HU, Muller U (2004). GlyR alpha3: an essential target for spinal PGE2-mediated inflammatory pain sensitization. Science.

[B75] Zeilhofer HU (2005). The glycinergic control of spinal pain processing. Cell Mol Life Sci.

[B76] Poisbeau P, Patte-Mensah C, Keller AF, Barrot M, Breton JD, Luis-Delgado OE, Freund-Mercier MJ, Mensah-Nyagan AG, Schlichter R (2005). Inflammatory pain upregulates spinal inhibition via endogenous neurosteroid production. J Neurosci.

[B77] Cummins TR, Dib-Hajj SD, Black JA, Waxman SG (2000). Sodium channels and the molecular pathophysiology of pain. Prog Brain Res.

[B78] Julius D, Basbaum AI (2001). Molecular mechanisms of nociception. Nature.

[B79] Lewin GR, Lu Y, Park TJ (2004). A plethora of painful molecules. Curr Opin Neurobiol.

[B80] Melzack R, Wall PD (1965). Pain mechanisms: a new theory. Science.

[B81] Game CJ, Lodge D (1975). The pharmacology of the inhibition of dorsal horn neurones by impulses in myelinated cutaneous afferents in the cat. Exp Brain Res.

[B82] Baba H, Ji RR, Kohno T, Moore KA, Ataka T, Wakai A, Okamoto M, Woolf CJ (2003). Removal of GABAergic inhibition facilitates polysynaptic A fiber-mediated excitatory transmission to the superficial spinal dorsal horn. Mol Cell Neurosci.

[B83] Furue H, Katafuchi T, Yoshimura M (2004). Sensory processing and functional reorganization of sensory transmission under pathological conditions in the spinal dorsal horn. Neurosci Res.

[B84] Seagrove LC, Suzuki R, Dickenson AH (2004). Electrophysiological characterisations of rat lamina I dorsal horn neurones and the involvement of excitatory amino acid receptors. Pain.

[B85] Buesa I, Ortiz V, Aguilera L, Torre F, Zimmermann M, Azkue JJ (2006). Disinhibition of spinal responses to primary afferent input by antagonism at GABA receptors in urethane-anaesthetised rats is dependent on NMDA and metabotropic glutamate receptors. Neuropharmacology.

[B86] McGowan MK, Hammond DL (1993). Intrathecal GABA(B) antagonists attenuate the antinociception produced by microinjection of L-glutamate into the ventromedial medulla of the rat. Brain Res.

[B87] Antal M, Petko M, Polgar E, Heizmann CW, Storm-Mathisen J (1996). Direct evidence of an extensive GABAergic innervation of the spinal dorsal horn by fibres descending from the rostral ventromedial medulla. Neuroscience.

[B88] Lin Q, Peng YB, Willis WD (1996). Inhibition of primate spinothalamic tract neurons by spinal glycine and GABA is reduced during central sensitization. J Neurophysiol.

[B89] Peng YB, Lin Q, Willis WD (1996). Effects of GABA and glycine receptor antagonists on the activity and PAG-induced inhibition of rat dorsal horn neurons. Brain Res.

[B90] Hao JX, Xu XJ, Aldskogius H, Seiger A, Wiesenfeld-Hallin Z (1991). Allodynia-like effects in rat after ischaemic spinal cord injury photochemically induced by laser irradiation. Pain.

[B91] Xu XJ, Hao JX, Aldskogius H, Seiger A, Wiesenfeld-Hallin Z (1992). Chronic pain-related syndrome in rats after ischemic spinal cord lesion: a possible animal model for pain in patients with spinal cord injury. Pain.

[B92] Woolf CJ, Bennett GJ, Doherty M, Dubner R, Kidd B, Koltzenburg M, Lipton R, Loeser JD, Payne R, Torebjork E (1998). Towards a mechanism-based classification of pain?. Pain.

[B93] Block BM, Hurley RW, Raja SN (2004). Mechanism-based therapies for pain. Drug News Perspect.

[B94] Smith PA (2004). Neuropathic pain: drug targets for current and future interventions. Drug News Perspect.

[B95] Woolf CJ (2004). Pain: moving from symptom control toward mechanism-specific pharmacologic management. Ann Intern Med.

[B96] Harden RN (2005). Chronic neuropathic pain. Mechanisms, diagnosis, and treatment. Neurologist.

[B97] Hines ML, Carnevale NT (1997). The NEURON simulation environment. Neural Comput.

[B98] Mainen ZF, Joerges J, Huguenard JR, Sejnowski TJ (1995). A model of spike initiation in neocortical pyramidal neurons. Neuron.

[B99] Traub RD, Miles R (1991). Neuronal Networks of the Hippocampus.

[B100] Prescott SA, De Koninck Y (2005). Integration time in a subset of spinal lamina I neurons is lengthened by sodium and calcium currents acting synergistically to prolong subthreshold depolarization. J Neurosci.

[B101] Mainen ZF, Sejnowski TJ (1996). Influence of dendritic structure on firing pattern in model neocortical neurons. Nature.

[B102] Dahlhaus A, Ruscheweyh R, Sandkuhler J (2005). Synaptic input of rat spinal lamina I projection and unidentified neurones in vitro. J Physiol.

[B103] Jahr CE, Stevens CF (1990). A quantitative description of NMDA receptor-channel kinetic behavior. J Neurosci.

[B104] Jahr CE, Stevens CF (1990). Voltage dependence of NMDA-activated macroscopic conductances predicted by single-channel kinetics. J Neurosci.

[B105] Destexhe A, Mainen ZF, Sejnowski TJ, Koch C and Segev I (1998). Kinetic models of synaptic transmission. Methods in Neuronal Modeling.

[B106] Furue H, Narikawa K, Kumamoto E, Yoshimura M (1999). Responsiveness of rat substantia gelatinosa neurones to mechanical but not thermal stimuli revealed by in vivo patch-clamp recording. J Physiol.

